# Genetic Analysis and QTL Detection on Fiber Traits Using Two Recombinant Inbred Lines and Their Backcross Populations in Upland Cotton

**DOI:** 10.1534/g3.116.031302

**Published:** 2016-06-23

**Authors:** Lianguang Shang, Yumei Wang, Xiaocui Wang, Fang Liu, Abdugheni Abduweli, Shihu Cai, Yuhua Li, Lingling Ma, Kunbo Wang, Jinping Hua

**Affiliations:** * Department of Plant Genetics and Breeding/Key Laboratory of Crop Heterosis and Utilization of Ministry of Education/Beijing Key Laboratory of Crop Genetic Improvement, China Agricultural University, Beijing 100193, China; † Institute of Cash Crops, Hubei Academy of Agricultural Sciences, Wuhan 430064, China; ‡ Institute of Cotton Research, Chinese Academy of Agricultural Sciences/State Key Laboratory of Cotton Biology, Anyang 455000, Henan, China

**Keywords:** fiber quality, QTL, recombinant inbred line, backcross population, Upland cotton

## Abstract

Cotton fiber, a raw natural fiber material, is widely used in the textile industry. Understanding the genetic mechanism of fiber traits is helpful for fiber quality improvement. In the present study, the genetic basis of fiber quality traits was explored using two recombinant inbred lines (RILs) and corresponding backcross (BC) populations under multiple environments in Upland cotton based on marker analysis. In backcross populations, no significant correlation was observed between marker heterozygosity and fiber quality performance and it suggested that heterozygosity was not always necessarily advantageous for the high fiber quality. In two hybrids, 111 quantitative trait loci (QTL) for fiber quality were detected using composite interval mapping, in which 62 new stable QTL were simultaneously identified in more than one environment or population. QTL detected at the single-locus level mainly showed additive effect. In addition, a total of 286 digenic interactions (E-QTL) and their environmental interactions [QTL × environment interactions (QEs)] were detected for fiber quality traits by inclusive composite interval mapping. QE effects should be considered in molecular marker-assisted selection breeding. On average, the E-QTL explained a larger proportion of the phenotypic variation than the main-effect QTL did. It is concluded that the additive effect of single-locus and epistasis with few detectable main effects play an important role in controlling fiber quality traits in Upland cotton.

Cotton is an important cash crop providing most of the natural fiber for the textile industry. Upland cotton (*Gossypium hirsutum* L.) accounts for 95% of the total production of cotton fiber in the world (Chen *et al.* 2007). Cotton fiber is widely employed in the textile industry. With the development of spinning technology, the improvement of fiber quality is becoming highly crucial in Upland cotton (Kohel *et al.* 2001). However, a major problem for cotton breeding is that fiber quality has a negative genetic correlation with cotton yield. The advent of molecular markers made it possible for cotton breeders to improve yield and fiber quality traits in cotton (Paterson *et al.* 1988). Previous studies have shown that fiber quality traits were quantitative traits and were affected by genetic background and the specific growing environment. These studies also observed that epistatic effects and genotype × environment (GE) interaction effects played an important role in the genetic basis of fiber traits (Paterson *et al.* 2003; Shen *et al.* 2006). Understanding of the genetic basis of both yield and fiber quality traits is vital for cotton breeding.

Detection of stable quantitative trait loci (QTL) for fiber quality is essential for developing cotton cultivars with superior fiber quality using molecular marker-assisted selection (MAS) strategy. A large number of studies on mapping main agronomic characters can be found in the CottonGen database (Yu *et al.* 2014). Hundreds of QTL conditioning fiber traits were obtained based on intraspecific genetic populations in Upland cotton (Said *et al.* 2015). These fiber QTL identified using low density genetic maps have very limited value in MAS programs. Fortunately, publically available sequences of the D_5_ genome of *G. raimondii*, A_2_ genome of *G. arboreum*, AD_1_ genome of *G. hirsutum*, and AD_2_ genome of *G. barbadense* provide an opportunity to improve the density of intraspecific genetic maps (Paterson *et al.* 2012; Wang *et al.* 2012; F. Li *et al.* 2014, 2015; Yuan *et al.* 2015; T. Zhang *et al.* 2015). Recently, a population of 178 recombinant inbred lines (RILs) was developed from a cross between *G. hirsutum*. acc ‘DH962’ and *G. hirsutum*. cv ‘Jimian5′. A total of 644 polymorphic loci were employed to construct a genetic linkage map, and a total of 64 QTL associated with fiber qualities were identified in seven environments (Wang *et al.* 2015a). Z. Zhang *et al.* (2015) developed a population of 196 RILs from a cross between ‘0-153’ and ‘sGK9708’ and detected a total of 37 QTL for fiber quality traits on chromosome 25, of which 17 were stably identified under at least two environments. Wang *et al.* (2015b) constructed an intraspecific genetic map of Upland cotton containing 1013 loci by developing markers using parental restriction site-associated DNA sequencing. And 27 new QTL for yield and fiber quality were identified, suggesting that the efficiency of QTL identification is greatly improved by the increase in genetic map density. Islam *et al.* (2016) validated three QTL regions controlling three fiber quality traits and further fine-mapped with 27 new single nucleotide polymorphism markers. The limitations of traditional breeding can be alleviated using an Upland cotton intraspecific high density genetic map to identify fiber QTL under multiple environments and further to conduct molecular marker-assisted breeding.

In our previous studies, two RIL populations and two corresponding backcross populations were applied to elucidate the genetic basis of oil content, seed index, and yield heterosis in Upland cotton (Shang *et al.* 2016a,b). In our laboratory, 39 QTL for fiber quality were identified in three generations (four environments) of F_2_, F_2: 3_ and F_2: 4_ populations derived from cross ‘GX1135’ × ‘GX100-2’ (Liang *et al.* 2013). Recently, 20 QTL associated with fiber quality traits were detected using 581 loci and a separate RIL population derived from a cross ‘GX1135’ × ‘GX100-2’. Among 20 QTL, four QTL were again detected, verifying the previous results in F_2_, F_2:3_, and F_2:4_ populations (Shang *et al.* 2015a). In the present research, two RIL populations and their backcross progeny derived from two hybrids were used to further detect the stable QTL for fiber quality traits in Upland cotton.

Previous reported results showed that most of the QTL controlling fiber quality traits were identified only in single mapping populations, such as F_2: 3_ (Mei *et al.* 2004; Liang *et al.* 2013) and RIL (Wang *et al.* 2015a; Shang *et al.* 2015a; Z. Zhang *et al.* 2015). There are few reports available of fiber quality QTL using multiple genetic populations in multiple environments. The objective of the present study is to detect stable QTL and conduct the genetic analysis of fiber quality traits by the single-locus and two-locus analysis using two RIL populations and their backcross progeny in Upland cotton. This study will provide new insights into the genetic basis of fiber quality and make contributions to improve fiber quality in Upland cotton.

## Materials and Methods

### Plant materials and population construction

As described previously (Shang *et al.* 2016a,b), two hybrids were used in the present research. One is ‘Xinza 1’ (Liang *et al.* 2013, 2015; Shang *et al.* 2015a,b, 2016c; hereinafter referred to as the ’XZ hybrid’), derived from ‘GX1135’ × ‘GX100-2’. The other one has a common female parent with ‘Xinza 1’, derived from ‘GX1135’ × ‘VGX100-2’ (Shang *et al.* 2016a,b; hereinafter referred to as the ‘XZV hybrid’).

In total, four populations were employed: (1) the RIL population of the XZ hybrid; (2) another RIL population (RILV) from the XZV hybrid; (3) a backcross population (BC) of the XZ hybrid (the BC population included 177 BCF_1_ hybrids, and each BCF_1_ hybrid was from a cross where one F_9_ RIL was used as the female parent and the common parent GX1135 was used as the male parent, respectively); and (4) another backcross population (BCV) of the XZV hybrid. One hundred and eighty BCVF_1_ hybrids were developed from crosses between RILs from the F_9_ RILV population used as the female parent and the common parent GX1135 used as the male parent, respectively (Shang *et al.* 2016a,b).

For ease of description, we refer to the RIL(V)s in BC(V) population as the RIL(V)′ population, respectively. In the BC(V) population, six-row plots were set which included the BCF_1_ hybrids RIL(V)′ × GX1135 in the middle, and its corresponding female RIL(V)′ and the recurrent parent, GX1135. Each line in the RIL(V)′ population was used as the female parent in the BC(V) population and was the same as that in the RIL(V) population. In BCF_1_ population experiments of population 3 (BC) and population 4 (BCV), each plot consisted of two rows of the female RIL(V)′, BCF_1_ hybrids and GX1135, respectively.

In addition, two special plots, each consisting of two rows of the XZ hybrid F_1_ and its parents, respectively, were used as controls in the experiment of population 1 and 3. Similar controls were set for experiments of population 2 and 4, and each plot consisted of the XZV hybrid F_1_ and its parents.

### Field trials and phenotypic evaluation

The parents together with the four populations were evaluated in three environments in China (Shang *et al.* 2015a): E1 – Quzhou Experimental Station of the China Agricultural University, Handan, Hebei Province; E2 – Guoxin Seed Company, Limited, Cangzhou, Hebei Province; and E3 – Xiangyang Academy of Agricultural Sciences, Xiangyang, Hubei Province. The field planting followed a randomized complete block design with duplicate at each location in 2012. Two-row plots were 80 cm and 50 cm row spacing alternately for the experiment in E1 and E2, and two-row plots in E3 were 100 cm and 80 cm row spacing alternately. The length of plots was 4 m in E1, and 3 m in E2 and E3. In the experiment for populations 1 and 2, two-row plots with each line were used. In the experiment for populations 3 and 4, six-row plots with each plot consisting of two rows of BCF_1_ hybrid [RIL(V) × GX1135], and two for each of the corresponding parents: the female RIL(V)′ and GX1135. Field management followed the local conventional standard field practices (Shang *et al.* 2016a,b).

Thirty open bolls from each plot were sampled by hand in three sites, respectively. Fiber quality traits were measured with an HVI 900 instrument (USTER HVISPECTRUM, SPINLAB) at the Cotton Fiber Quality Inspection and Test Center of the Ministry of Agriculture (Anyang, China). The fiber quality traits included 2.5% fiber span length (mm), fiber uniformity (%), fiber strength (cN/tex), fiber elongation, and micronaire.

### Genotype analysis

Extraction of individual genomic DNA and population genotype analysis were carried out following the methods of Liang *et al.* (2013). A total of 48,836 pairs of SSR primer were employed to screen polymorphic loci between three parents (Shang *et al.* 2016a). The SSR primers newly added in this study included the EST-SSR primers, named as CAU primers, developed from the salt-tolerance EST sequences, SWU and PGML primers developed from the *G. raimondii* genome sequence, and ICR primers developed from the *G. arboreum* genome sequence. In total, 653 polymorphic loci for the XZ hybrid and 400 polymorphic loci for the XZV hybrid were used to conduct genotype analysis of two RIL populations (Shang *et al.* 2016a,b). The genotype for each BCF_1_ was deduced on the basis of the RIL genotype used as the parent for the cross.

### Data analysis

A basic statistical analysis was implemented using the software SPSS version 19.0 (SPSS, Chicago). The software MAPMAKER 3.0 was employed to construct a genetic linkage map with the *Kosambi* function (Lander *et al.* 1987). For the RIL(V) and RIL(V)′ populations, the means from two replications were used as raw data at each location. For each of the BC(V)F_1_ populations, the means of the BC(V)F_1_s were used independently as raw data at three locations. Single-locus QTL was conducted using composite interval mapping by the software WinQTL Cartographer 2.5 in RIL(V)′, RIL(V), and BC(V)F_1_ data (Zeng 1994; Wang *et al.* 2005). A LOD threshold of 3.0 was used to declare suggestive QTL, whereas the same QTL in another environment or population with LOD of at least 2.0 was considered to be a common QTL (Shang *et al.* 2015a). The graphic representation of the linkage group was created using the software Map Chart 2.2 (Voorrips 2002). QTL nomenclature used in rice was employed in the present study (McCouch *et al.* 1997). Two-locus analysis that tests the main-effect QTL (M-QTL), and digenic epistatic QTL (E-QTL) and their environmental interactions (QTL × environment, QE), was conducted using the software ICIMapping 4.0 (www.isbreeding.net). LOD thresholds were respectively set at 2.5 and 5.0 for declaring the presence of M-QTL, E-QTL, and their QEs (S. Li *et al.* 2015).

### Data availability

All raw data are available as Supporting Information Table S9 and Table S10, which include genotypes and traits of two hybrids.

## Results

### Performance of fiber quality traits

The means of phenotypic data for fiber quality traits of two hybrids in three environments are shown in Table 1. Not all the fiber quality traits possessed higher phenotype values in heterozygotes (BCF_1_s) than in respective homozygotes (RILs). Most of the extreme lines in the RIL(V) populations exceeded those of BC(V)F_1_ populations under different environments. The analysis of variance in RIL(V) and BC(V) populations was conducted and significant genotypic variances and environment variances for most of the fiber quality traits were found in four populations (Table 2). Significant variations for fiber quality traits are observed in two hybrids. Skewness and kurtosis values were calculated, and results showed that all fiber quality traits fit a normal distribution in two RIL and two backcross populations of two hybrids (data not shown). The BCF_1_ population had higher means for most of the fiber quality traits than the RIL population in the two hybrids. These results suggested that fiber quality traits of RIL(V) and BC(V) populations were highly variable and conducive for QTL analysis.

**Table 1:** Summary statistics on fiber quality traits in two hybrids

	Mean		Min		Max		Parents	
Trait	RIL	BC	RIL	BC	RIL	BC	♀	♂
XZ hybrid																	
Fiber length (mm)	28.48	28.52	25.96	26.74	31.01	30.49	28.72	27.78
Fiber uniformity	84.63	84.88	81.82	82.22	86.78	87.08	84.28	85.00
Fiber strength (cN/tex)	29.12	29.29	25.55	27.13	32.38	31.78	29.75	27.90
Fiber elongation	6.91	6.77	6.43	6.50	7.28	7.00	6.83	6.68
Micronaire	4.61	4.68	3.59	4.08	5.42	5.25	4.76	4.74
XZV hybrid								
Fiber length (mm)	28.07	28.60	24.81	26.09	31.86	31.38	28.72	28.47
Fiber uniformity	84.45	85.25	81.40	82.02	86.50	87.25	84.28	83.97
Fiber strength (cN/tex)	29.75	30.18	25.62	27.33	33.80	32.77	29.75	30.14
Fiber elongation	6.72	6.67	6.30	6.40	7.10	6.93	6.83	6.73
Micronaire	4.39	4.28	3.27	3.64	5.31	4.86	4.76	4.11

**Table 2:** The results of analysis of variance (ANOVA) of fiber quality traits

		RIL	BCF_1_	RILV	BCVF_1_
Trait	Source of Variation	MS	MS	MS	MS
Fiber length	G	4.43**	1.73**	9.44**	2.71**
	E	1259.77**	998.88**	686.68**	719.22**
	G × E	0.88**	0.72	1.19**	1.00
	e	0.67	0.82	0.90	0.91
Fiber uniformity	G	1.91*	1.85*	2.65**	1.44
	E	967.44**	839.95**	1084.62**	838.20**
	G × E	1.61	1.44	1.47	1.79
	e	1.46	1.45	1.39	1.71
Fiber strength	G	6.26**	2.16**	10.76**	3.36**
	E	775.17**	119.33**	38.46**	65.35**
	G × E	1.33	1.25	2.58**	1.43
	e	1.22	1.37	1.67	1.57
Fiber elongation	G	0.07**	0.02	0.08**	0.02
	E	0.09*	6.03**	1.21**	21.12**
	G × E	0.03*	0.02	0.04*	0.02
	e	0.02	0.03	0.03	0.02
Micronaire	G	0.44**	0.15**	0.57**	0.19**
	E	35.80**	66.45**	59.14**	100.40**
	G × E	0.11**	0.07	0.10	0.08
	e	0.07	0.06	0.08	0.07

* *P* = 0.05, **P = 0.01. G, genotype; E, environment; e, error MS, mean square.

### Correlation analysis among fiber quality traits

Correlation analysis was carried out using the mean values of three environments, respectively (Table 3). The majority of fiber quality traits were significantly associated with each other in two hybrids. Fiber length is significantly positively correlated with fiber strength and fiber elongation, but was negatively correlated with micronaire. Fiber strength was significantly positively correlated with fiber elongation, but it was negatively correlated with micronaire. Fiber uniformity was significantly positively correlated with fiber elongation. These results were consistent with a previous report (Shang *et al.* 2015a). The correlation coefficients among the mean values of RILs and their BCF_1_s for fiber quality traits are shown in Supplemental Material, Table S1. Most of the fiber trait values of the RILs and that of their BCF_1_s showed significant positive correlation. Population performance of the BCF_1_ for most of the fiber traits was largely determined by performance of the parental RIL.

**Table 3:** Correlations between fiber quality traits of RIL and backcross populations in two hybrids

		Fiber Length		Fiber Uniformity		Fiber Strength		Fiber Elongation	
Trait	Env.	RIL	BC	RIL	BC	RIL	BC	RIL	BC
XZ hybrid									
Fiber uniformity	E1	0.27**	0.28**						
	E2	0.05	0.04						
	E3	0.15	−0.05						
Fiber strength	E1	0.64**	0.51**	0.21**	0.21**				
	E2	0.76**	0.58	0.10	0.05				
	E3	0.58**	0.38**	0.32**	0.17*				
Fiber elongation	E1	0.60**	0.57**	0.15*	0	0.63**	0.64**		
	E2	—	—	—	—	—	—		
	E3	0.52**	0.43**	0.17*	0.18*	0.44**	0.59**		
Micronaire	E1	−0.27**	−0.09	0.33**	0.11	−0.22**	−0.25**	0.04	0.07
	E2	−0.25**	−0.12	−0.04	0.13	−0.15*	−0.13	—	—
	E3	−0.48**	−0.34**	−0.09	0.1	−0.34**	−0.16*	0.03	0.08
XZV hybrid									
Fiber uniformity	E1	0.30**	0.37**						
	E2	0.17	0						
	E3	0.12	−0.01						
Fiber strength	E1	0.65**	0.40**	0.42**	0.33**				
	E2	0.73	0.65	0.31	0.14				
	E3	0.53**	0.32**	0.27**	0.14				
Fiber elongation	E1	0.68**	0.49**	0.33**	0.29**	0.77**	0.78**		
	E2	—	—	—	—	—	—		
	E3	0.49**	0.58**	0.11	−0.02	0.60**	0.43**		
Micronaire	E1	−0.31**	−0.05	0.12	0.23**	−0.07	0.08	0.16*	0.18*
	E2	−0.18	−0.18	−0.02	−0.03	−0.12	−0.16	—	—
	E3	−0.57**	−0.34**	0	0.11	−0.33**	−0.40**	0.01	0.04

* and ** indicate that the correlation is significant at 0.05 and 0.01 probability levels, respectively. — indicates missing data. Env., environment; E1, Handan; E2, Cangzhou; E3, Xiangyang.

### QTL analysis for fiber quality traits at the single-locus level

Two genetic linkage maps were previously constructed based on the polymorphic loci identified in two hybrids (Shang *et al.* 2016a,b). For the XZ hybrid, the genetic map with 623 loci spanned 3889.9 cM. For the XZV hybrid, the genetic map with 308 loci spanned 3048.4 cM. QTL detected using composite interval mapping for fiber quality traits in XZ and XZV hybrids are shown in Table S2 and Figure S1. A total of 71 and 40 QTL were detected for fiber quality traits in RIL(V), RIL(V)′, and BC(V)F_1_ data sets of XZ and XZV hybrids in three environments, respectively. The genetic effect identified in the RIL population is generally larger than in the backcross population. These results suggested that heterozygosity was not always necessarily advantageous for the expression of the fiber quality traits.

In the XZ hybrid, a total of 17 QTL were detected for fiber length (FL) in three data sets, among which 13, 12, and 9 QTL were identified for FL in the RIL′s, RILs, and BCF_1_ hybrids data sets, respectively. Thirteen QTL were identified in more than two environments or populations. Two QTL, *qFL-Chr5-2* and *qFL-Chr21-1*, were identified simultaneously in three environments and populations, which were different genetic effects. In the XZV hybrid, a total of 10 QTL were detected for FL in three data sets, among which seven, seven, and five QTL were respectively identified for FL in the RILV′s, RILVs, and BCVF_1_ data sets. Eight QTL were identified in more than two environments or populations. Two QTL, *qFL-Chr2-2* and *qFL-Chr14-1*, with the same genetic direction were identified simultaneously in three environments and populations.

For fiber uniformity (FU), in the XZ hybrid, a total of six QTL were resolved explaining from 4.33% to 14.18% of phenotypic variance (PV). Two QTL were identified in more than two environments or populations. In the XZV hybrid, three QTL were detected and one QTL was identified in two populations.

For fiber strength (FS), in the XZ hybrid, a total of 15 QTL were detected in three data sets, among which 11, eight, and six QTL were respectively identified in the RIL′s, RILs, and BCF_1_ hybrids data. Eleven QTL were identified in more than two environments or populations. In the XZV hybrid, a total of eight QTL were detected, among which two, six, and five QTL were identified in the RILV′s, RILVs, and BCVF_1_ hybrids data, respectively. Four QTL were identified in more than two environments or populations, in which *qFS-Chr26-1* with negative genetic effect was stably expressed in multitude environments and populations.

For fiber elongation (FE), in the XZ hybrid, a total of 11 QTL were detected in three data sets, among which five, four, and six QTL were identified in the RIL′s, RILs, and BCF_1_ data, respectively. Three QTL were identified in more than two environments or populations. In the XZV hybrid, a total of six QTL were detected, among which three, five, and one QTL were respectively identified in the RILV′s, RILVs, and BCVF_1_ data. Two QTL were identified in more than two environments or populations.

For fiber micronaire (FM), in the XZ hybrid, a total of 21 QTL were resolved explaining from 3.76% to 25.93% of PV. Eleven QTL were identified in more than two environments or populations, among which two stable, QTL *qFM-Chr2-1* and *qFM-Chr19-1*, were simultaneously observed in two environments and three populations. In the XZV hybrid, 13 QTL were detected and seven QTL were identified in more than two environments or populations, among which one major QTL, *qFM-Chr14-1*, was observed in all of the environments and populations explaining from 3.97% to 14.06% of PV.

### QTL and QE interactions resolved by two-locus analyses

A total of 98 and 88 M-QTL and QEs were respectively detected by inclusive composite interval mapping (ICIM) in five fiber quality traits of XZ and XZV hybrids (Table 4, Table S3, and Table S4). In the XZ hybrid, a total of 65 and 33 M-QTL and QEs were detected in the RILs and BCF_1_ hybrids data, respectively. On average, M-QTL explained 2.35% and 2.14% of the phenotype variation, and the QE explained 0.74% and 0.86% of the phenotype variation in the RILs and BCF_1_ hybrids data, respectively. In the XZV hybrid, a total of 56 and 32 M-QTL and QEs were detected in the RILVs and BCVF_1_ hybrids, respectively. On average, M-QTL explained 2.73% and 1.77% of the phenotype variation, and the QE explained 0.43% and 0.92% of the phenotype variation in the RILVs and BCVF_1_ hybrids data, respectively.

**Table 4:** Summary of M-QTL and E-QTL detected controlling fiber quality traits by ICIM in two hybrids

Trait		RIL			BCF_1_			RILV			BCVF_1_	
M-QTL	*n*	P(A)	P(AE)	*n*	P(A)	P(AE)	*n*	P(A)	P(AE)	*n*	P(A)	P(AE)
Fiber length	12	2.67	0.58	9	2.17	0.72	18	2.94	0.13	7	2.18	0.59
Fiber uniformity	8	1.06	1.20	8	1.35	1.12				6	0.31	1.74
Fiber strength	21	2.41	0.41	7	2.20	0.76	9	3.20	0.42	5	2.83	0.56
Fiber elongation	8	2.80	1.17	2	3.17	0.86	6	2.17	0.90			
Micronaire	16	2.79	0.36	7	1.80	0.87	23	2.61	0.25	14	1.78	0.78
Mean	13	2.35	0.75	6.6	2.14	0.86	11.2	2.73	0.43	6.4	1.77	0.92
												
E-QTL	*n*	P(AA)	P(AAE)	*n*	P(AA)	P(AAE)	*n*	P(AA)	P(AAE)	*n*	P(AA)	P(AAE)
Fiber length	20	3.38	0.32	16	2.97	1.23	34	4.13	0.17	13	2.89	0.60
Fiber uniformity	6	2.28	1.53	5	1.76	1.74	6	2.69	0.90			
Fiber strength	14	2.98	0.32	7	1.98	1.31	25	3.30	0.42	7	3.13	0.47
Fiber elongation	6	4.65	0.46	1	1.82	4.27	1	6.43	0.24			
Micronaire	44	3.69	0.46	38	2.74	0.96	35	3.66	0.15	8	2.52	0.85
Mean	18	3.40	0.61	13.4	2.25	1.90	20.2	4.04	0.38	5.6	2.85	0.64

*n*, the number of QTL identified. P (in %) was the mean of trait PVs explained by a single M-QTL or E-QTL.

In total, 157 and 129 E-QTL and QEs were detected by ICIM in five fiber quality data sets of XZ and XZV hybrids, respectively (Table 4, Table S5, and Table S6). In the XZ hybrid, a total of 90 and 67 E-QTL and QEs were detected in the RILs and BCF_1_ hybrids data, respectively. On average, E-QTL explained 3.40% and 2.25% of the phenotype variation, and the QE explained 0.61% and 1.90% of the phenotype variation in the RILs and BCF_1_ hybrids data, respectively. In the XZV hybrid, a total of 101 and 28 E-QTL and QEs were detected in the RILVs and BCVF_1_ hybrids data, respectively. On average, E-QTL explained 4.04% and 2.85% of the phenotype variation, and the QE explained 0.38% and 0.64% of the phenotype variation in the RILVs and BCVF_1_ hybrids data, respectively.

## Discussion

### Advantage of permanent RIL and BC_1_ populations design

Fiber quality traits are quantitatively inherited, and the QTL detected tend to vary under different environments. In order to identify more stable and convincing QTL in more than one environment, permanent populations such as RILs are required. Permanent populations possessing heterozygotes could be applied to study the genetic basis of complex traits using QTL mapping strategy. For example, BC_1_ populations based on RIL population were previously constructed and used to conduct QTL analysis referring to complex agronomic traits (Mei *et al.* 2005; You *et al.* 2006, Jiang *et al.* 2014; Shang *et al.* 2016b). In this study, we constructed the BC_1_ population using the RIL population to identify QTL contributing to fiber quality traits and estimate their genetic effects in Upland cotton. Several QTL that were not identified in the RIL population can be detected in the BCF_1_ population, for example, *qFL-Chr2-2* was identified only in the BCF_1_ population in the XZ hybrid. Moreover, the QTL identified using the RIL population could be verified using the BCF_1_ population in the current study. For instance, the QTL *qFM-Chr19-1* identified in RIL populations was confirmed in the BCF_1_ population in both environments again in the XZ hybrid. Furthermore, the QTL detected in the XZ hybrid could be verified in the XZV hybrid in that two hybrids shared one parent (Shang *et al.* 2016b). The advantages of the permanent BC_1_ population are that it can be repeatedly made by RILs and the genotype of the BC_1_ population can be easily deduced by the genotype of the RIL population. In addition, the QTL actions can be inferred by comparing the genetic effects of RIL, BC_1_ performance, and midparental heterosis (Mei *et al.* 2005). However, the disadvantage of the BC_1_ population is that only half of the possible heterozygous loci are available (Radoev *et al.* 2008). It may be the reason why some QTL with dominant effect are omitted using BCF_1_ data rather than F_2_ population data.

### Consensus QTL and improvement of fiber quality

Two RIL populations and two corresponding backcross populations were developed and used to detect stable QTL in the present research. In a previous study, 19 QTL including eight for FL, three for FS, four for FE, and four for FM were detected in the RIL population derived from Upland cotton cross ‘GX1135’ × ‘GX100-2’ (Table S7). These 19 QTL for fiber traits detected previously were once again identified in the current study. In XZ and XZV hybrids, 62 stable QTL were identified in more than one environment or population. A stable QTL, *qFS-Chr26-1*, flanked by HAU1571 and PGML2562 was detected in RILV′, RILV, and BCV populations in three environments, and this QTL could contribute to 6.07–15.03% of the phenotypic variation. Another stable QTL, *qFM-Chr14-1*, was detected in RILV′, RILV, and BCV populations, explaining the phenotypic variation by 3.97–14.06%, respectively. The single-locus QTL identified by composite interval mapping and the M-QTL from ICIM were compared. A total of 59 common QTL were found, and common QTL are shown as blue figures in Table S2. In addition, QTL mapping for fiber quality using the overall means across three environments (joint analysis) were conducted, and a total of 53 QTL for fiber quality traits were identified (Table S8). Of 53 QTL, 44 QTL detected using joint analysis were the same as the QTL with a single environment. These novel stable QTL for fiber quality traits identified using multiple populations and environments will be helpful to improve fiber quality in the future. We compared the QTL detected in the current study with QTL identified in other studies, however the same QTL were not found. It is difficult to search for common QTL, because the genetic map, population types, population structure, and environmental conditions vary and affect the comparison of common QTL (Shang *et al.* 2015a).

The result of two hybrids can be compared and verified using the common markers located in two connected genetic maps. Two consistent QTL were acquired in both hybrid populations. A good example is the QTL *qFL-Chr2-1* for FL on chromosome 2 in the XZV hybrid; furthermore, we also identified another two QTL, *qFL-Chr2-1* and *qFL-Chr2-2*, for FL in the XZ hybrid. These two QTL identified in the XZ hybrid were located in the same marker region as mentioned above and had a narrower interval of flanking markers than that in the XZV hybrid. Therefore, using connected genetic populations may have increased the number of common QTL identified and improved the accuracy of QTL location (Blanc *et al.* 2006).

It is difficult to simultaneously improve yield and fiber quality in Upland cotton breeding programs (Liang *et al.* 2013). In the present study, QTL that were located on chromosome five possessed multiple effects on yield and fiber quality. Two stable QTL with flanking markers between SWU20917 and NAU6240 associated with FL and FS shared a common marker interval with the QTL referring to the boll weight and lint percentage (Shang *et al.* 2016b). These stable QTL simultaneously controlled yield and fiber quality with a large contribution to the phenotypic variation and provided valuable information for pyramiding elite genes of yield and fiber quality traits. Further study should be conducted to prove the effects of these QTL in the improvement of yield and fiber quality in Upland cotton.

### Genetic basis of fiber quality traits and QTL × environment interaction

The genetic basis of fiber quality traits is explored using two connected RIL populations and two corresponding BCF_1_ populations at the single-locus and two-locus levels in Upland cotton. The number and phenotypic variation of QTL identified using composite interval mapping in the RIL population were collectively larger than those QTL detected in the BCF_1_ population in the XZ and XZV hybrids. This suggested that the QTL controlling fiber quality traits mainly showed an additive effect at the single-locus level.

At the two-locus level, lots of digenic interactions and QEs resolved by ICIM were acquired in two hybrids. The epistasis detected had been previously classified into three types: (I) two loci with significant M-QTL, (II) one locus with M-QTL and another locus without significant M-QTL, and (III) two loci without significant M-QTL (Li *et al.* 2001; Shang *et al.* 2016d). In the XZ and XZV hybrids, we found that 39 of 301 (12.96%) epistatic interactions were type II, and the remaining 262 (87.04%) were type III for fiber quality traits. No type I interactions were acquired (Table 5). The fact that the digenic interaction mainly included loci without detectable M-QTL further confirmed the importance of epistasis in Upland cotton breeding (Shen *et al.* 2006). In addition, the number of E-QTL and the mean of PVs explained by E-QTL for most of the fiber traits are much greater than that for M-QTL in two hybrids (Table 4). These results revealed that epistasis played an important role not only in the variation of the performance of the RIL(V) population but also in the expression of hybrids in the BC(V) population. These results are in agreement with previous results which indicated that additive effect and epistatic interaction are common and important for fiber quality traits in Upland cotton. Selection for improving trait values should pay attention to the best multilocus combinations and major loci (Shen *et al.* 2006).

**Table 5:** Type of epistatic interactions detected for fiber quality traits in two hybrids

		Type of Epistasis							
		I		II		III		Sum	
Trait	Data Set	XZ	XZV	XZ	XZV	XZ	XZV	XZ	XZV
Fiber length	RIL	0	0	1	3	19	31	20	34
	BC	0	0	0	2	16	11	16	13
Fiber uniformity	RIL	0	0	0	0	6	6	6	6
	BC	0	0	0	0	5	0	5	0
Fiber strength	RIL	0	0	2	2	12	23	14	25
	BC	0	0	3	2	13	11	16	13
Fiber elongation	RIL	0	0	1	0	5	1	6	1
	BC	0	0	0	0	1	0	1	0
Micronaire	RIL	0	0	8	10	36	25	44	35
	BC	0	0	4	1	34	7	38	8
Sum		0	0	19	20	147	115	166	135

Type of epistasis: (I) two loci with main-effect QTL, (II) a locus with main-effect QTL and a locus without significant main-effect QTL, and (III) two loci without significant main-effect QTL. Sum, total number of epistatic interactions.

The genotype × environment interactions accounted for a small proportion of the mean phenotypic variation for fiber traits (Table 4). The QTL for fiber quality in the BC(V) population was more sensitive to the environment than that in the RIL(V) population as shown by the mean phenotypic variation explained by QEs. Particular environmental conditions were important in the expression of fiber quality, especially for Upland cotton hybrid. The results suggest that trials in multiple environments are a prerequisite for evaluating fiber quality traits (Shang *et al.* 2016a). The environmental factors in QTL associated with fiber quality and epistasis, and effect of QEs should be considered in MAS breeding (Xing *et al.* 2002; Shang *et al.* 2016b).

Overall, the single-locus with additive effect and epistasis with few detectable main effects play an important role in controlling expression of fiber quality in Upland cotton.

## Supplementary Material

Supplemental Material
