# Supplementary material for: Genetic Analysis and QTL Detection on Fiber Traits Using Two Recombinant Inbred Lines and Their Backcross Populations in Upland Cotton
Source: G3 (Bethesda). 2016 Jun 23;6(9):2717–24. doi: 10.1534/g3.116.031302 (PMC5015930; doi:10.1534/g3.116.031302)
Supplement: Supplemental Material [file supp_6_9_2717__index.html]

Genetic Analysis and QTL Detection on Fiber Traits Using Two Recombinant Inbred Lines and Their Backcross Populations in Upland Cotton — Supplemental Material 

# Genetic Analysis and QTL Detection on Fiber Traits Using Two Recombinant Inbred Lines and Their Backcross Populations in Upland Cotton

## Supplemental Material for Shang *et al.*, 2016

**Files in this Data Supplement:**

- Figure S1 - Locations of QTLs controlling fiber quality traits identified in two hybrids. (.pdf, 970 KB)
- Table S1 - Correlations between RIL and BCF1 data sets in two hybrids. (.pdf, 14 KB)
- Table S2 - QTLs identified for fiber traits of RIL', RIL, and BCF1 populations by composite interval mapping in two hybrids. (.pdf, 683 KB)
- Table S3 - Main effects and environmental interactions detected for fiber quality traits in RIL and RILV populations by ICIMapping 4.0. (.pdf, 223 KB)
- Table S4 - Main effects and environmental interactions detected for fiber quality traits in BCF1 and BCVF1 populations by ICIMapping 4.0. (.pdf, 120 KB)
- Table S5 - Epistatic effects and environmental interactions detected for fiber quality traits in RIL and RILV populations using two-locus analysis by ICIMapping 4.0. (.pdf, 368 KB)
- Table S6 - Epistatic effects and environmental interactions detected for fiber quality traits in BCF1 and BCVF1 populations using two-locus analysis by ICIMapping 4.0. (.pdf, 208 KB)
- Table S7 - Same QTLs for fiber quality traits identified using composite interval mapping in previous RIL population (Shang *et al*. 2015a). (.pdf, 35 KB)
- Table S8 - QTLs identified for fiber traits using the overall means by composite interval mapping. (.pdf, 103 KB)
- Table S9 - Genotypes and traits of XZ hybrid used in this work. (.xls, 1952 KB)
- Table S10 - Genotypes and traits of XZV hybrid used in this work. (.xls, 1389 KB)
