# Supplementary material for: Genetic Analysis and QTL Detection on Fiber Traits Using Two Recombinant Inbred Lines and Their Backcross Populations in Upland Cotton
Source: G3 (Bethesda). 2016 Jun 23;6(9):2717–24. doi: 10.1534/g3.116.031302 (PMC5015930; doi:10.1534/g3.116.031302)
Supplement: Supplemental Material [file supp_g3.116.031302_TableS1.pdf]

Table S1 Correlations between RIL and BC data sets in two hybrids

Trait	Env.	Between RILs and BC	
		XZ hybrid	XZV hybrid
Fiber length	E1	0.44**	0.48**
	E2	0.26**	0.50**
	E3	0.41**	0.59**
Fiber uniformity	E1	-0.04	0.18*
	E2	0.10	0.02
	E3	0.10	0.17*
Fiber strength	E1	0.37**	0.44**
	E2	0.32**	0.41**
	E3	0.35**	0.50**
Fiber elongation	E1	0.31**	0.34**
	E2	-	-
	E3	0.19*	0.28**
Micronaire	E1	0.43**	0.41**
	E2	0.41**	0.47**
	E3	0.45**	0.52**

*, ** indicate that the correlation is significant at 0.05 and 0.01 probability levels, respectively
