# Supplementary material for: Genetic Analysis and QTL Detection on Fiber Traits Using Two Recombinant Inbred Lines and Their Backcross Populations in Upland Cotton
Source: G3 (Bethesda). 2016 Jun 23;6(9):2717–24. doi: 10.1534/g3.116.031302 (PMC5015930; doi:10.1534/g3.116.031302)
Supplement: Supplemental Material [file supp_g3.116.031302_TableS2.pdf]

Table S2 QTLs identified for fiber traits of RIL′, RIL, and BC populations by composite interval mapping in two hybrids

Trait	QTL	Env	Marker interval		RIL′			RIL			BC		
					LOD	A	Var%	LOD	A	Var%	LOD	A+D	Var%
					**XZ hybrid**								
FL	*qFL-Chr2-1*	E2	SWU12393	SWU11013	5.72	0.40	11.06						
	qFL-Chr2-2	E2	SWU12025	SWU11889							2.38	0.24	6.92
		E3	SWU12025	SWU11889							2.10	0.15	6.30
	qFL-Chr2-3	E3	SWU11976	SWU11950				2.31	0.20	5.05			
		E1	TMB1268	JESPR304	4.70	0.25	8.04						
	qFL-Chr5-1	E2	SWU20913	Gh260	2.27	0.29	5.89						
		E3	PGML0120	SWU20914				2.94	0.21	5.61			
	qFL-Chr5-2	E3	SWU20917	NAU6240	5.34	0.28	9.00						
		E2	SWU20917	NAU6240				3.95	0.39	11.03			
		E1	NAU6240	PGML1671				2.91	0.27	7.70			
		E3	PGML1671	PGML1917							3.74	0.16	7.20
	qFL-Chr5-3	E1	PGML1917	SWU17715							3.12	0.29	21.09
	qFL-Chr5-4	E1	NAU4034	SWU17713							4.30	0.19	8.57
		E1	NAU4034	SWU17713	8.94	0.37	16.39						
		E1	NAU4034	SWU17713				6.39	0.34	11.68			
		E2	NAU4034	SWU17713				2.90	0.29	5.64			
	qFL-Chr5-5	E3	PGML4457	MUSS193							3.15	0.15	6.24
		E3	PGML4457	MUSS193	4.18	0.25	7.92						
		E3	PGML4457	MUSS193				4.40	0.27	8.97			
	qFL-Chr5-6	E3	CGR5025	NBRI0694							4.72	0.18	9.11
		E3	CGR5025	NBRI0694	9.13	0.35	15.75						
		E3	NBRI0694	DPL0022				4.83	0.27	9.47			
	qFL-Chr5-7	E3	SWU17787	SWU13378	6.16	0.34	15.23						
		E3	SWU17787	SWU13378				3.62	0.29	10.37			
		E1	SWU17787	SWU13378	2.66	0.25	7.14						
	qFL-Chr9-1	E3	SWU15517	SWU15511	2.02	-0.15	3.00						
		E1	SWU15511	SWU15413	2.70	-0.19	4.50						
	qFL-Chr10-1	E1	CGR5873	ICR00093							3.12	-0.16	6.12
	qFL-Chr10-2	E1	ICR00093	ICR07050	3.66	-0.26	8.74						
	qFL-Chr10-3	E2	SWU20260	Gh144				3.89	-0.33	7.64			
		E1	Gh320	HAU0635				2.46	-0.22	5.31			
		E1	Gh320	HAU0635	5.45	-0.33	13.47						
	qFL-Chr18-1	E1	SWU22192	DPL0864				3.70	-0.25	6.53			
		E2	SWU22187	DC40150				2.07	-0.26	4.84			
	qFL-Chr19-1	E1	SWU14431	SWU17782				2.89	0.28	8.68			
		E3	SWU14431	SWU17782	4.36	0.27	9.01						
		E3	SWU17782	DPL0056							2.10	0.14	5.56
	qFL-Chr21-1	E1	SWU15915	SWU0189				2.32	-0.20	4.14			
		E2	SWU15915	SWU0189							2.16	-0.21	4.77
		E2	SWU0189	DPL0050a	3.81	-0.32	7.17						
		E3	SWU0189	DPL0050a	2.83	-0.20	5.14						

Trait	QTL	Env	Marker interval		RIL′			RIL			BC		
					LOD	A	Var%	LOD	A	Var%	LOD	A+D	Var%
		E3	SWU0189	DPL0050a							2.29	-0.13	4.60
	qFL-Chr21-2	E1	BNL3171	CGR5808				3.96	-0.27	7.67			
FU	*qFU-Chr2-1*	E3	SWU12025	SWU11889	3.54	-0.23	9.97						
	qFU-Chr2-2	E1	TMB2386	SWU12343							2.20	0.28	8.57
		E1	TMB2386	SWU12343	3.41	0.43	14.18						
	qFU-Chr2-3	E1	NBRI0014	SWU12107							2.25	0.20	4.33
		E3	NBRI0014	SWU12107				2.83	0.21	6.14			
	qFU-Chr3-1	E2	HAU2424	CER0028				3.03	-0.28	6.63			
	qFU-Chr10-1	E2	SWU20260	Gh144							3.61	-0.29	7.58
	qFU-Chr26-2	E1	C2_0135	PGML2321							3.38	-0.27	8.32
FS	*qFS-Chr1-1*	E1	ICR03725	SWU10987				4.01	-0.39	7.36			
	qFS-Chr1-2	E3	NAU3384	CGR5663							4.44	0.23	9.49
	qFS-Chr2-1	E1	NBRI0014	SWU12107							2.12	0.20	4.28
		E1	MGHES24	ICR11064	2.15	0.26	3.75						
	qFS-Chr4-1	E1	NAU2701	SWU18876				2.63	0.32	4.74			
		E2	SWU12672	HAU1332							3.41	-0.27	7.46
	qFS-Chr5-1	E3	SWU20917	NAU6240	6.03	0.38	14.08						
		E2	SWU20917	NAU6240				4.73	0.43	12.58			
		E3	SWU20917	NAU6240				4.91	0.34	11.57			
		E1	NAU6240	PGML1671				5.08	0.48	11.07			
	qFS-Chr5-2	E1	NAU4034	SWU17713							2.22	0.21	4.41
		E1	NAU4034	SWU17713	5.17	0.40	9.40						
	qFS-Chr11-1	E3	BNL3442b	ICR01810	3.43	-0.30	8.84						
	qFS-Chr13-1	E1	NAU3468	SWU22309	3.13	-0.33	6.14						
		E2	SWU22309	SWU22324	2.72	-0.36	6.75						
	qFS-Chr13-2	E2	DPL0308	DPL0535				2.23	-0.27	4.95			
		E2	DPL0535	CER0165	4.11	-0.38	7.95						
	qFS-Chr15-1	E3	CGR6889	DPL0182				3.06	0.24	5.64			
		E2	CGR6889	DPL0182				2.76	0.29	5.48			
	qFS-Chr18-1	E3	CIR099	NAU748	2.67	0.22	4.72						
		E1	CIR099	NAU748				3.38	0.36	6.34			
	qFS-Chr19-1	E1	NAU3437	NAU2894	3.33	0.33	6.10						
		E3	NAU3437	NAU2894	4.11	0.28	7.66						
		E1	NAU3437	NAU2894				2.49	0.31	4.52			
		E3	NAU3437	NAU2894				4.61	0.40	15.54			
	qFS-Chr21-1	E2	SWU14431	SWU15915				2.24	-0.27	4.73			
		E1	SWU15915	SWU0189	3.20	-0.32	5.87						
		E2	SWU0189	DPL0050a							2.16	-0.21	4.72
		E2	SWU0189	DPL0050a	6.65	-0.53	15.51						
	qFS-Chr21-2	E1	BNL3171	CGR5808							2.89	-0.24	5.80
		E1	BNL3171	CGR5808	3.98	-0.36	7.10						
		E2	BNL3171	CGR5808	4.52	-0.43	10.60						
	qFS-Chr26-1	E2	SWU18697	PGML1289	3.89	0.37	7.66						
FE	***qFE-Chr2-1***	E1	DPL0217	SWU12025	5.37	0.05	10.46						

Trait	QTL	Env	Marker interval		RIL′			RIL			BC		
					LOD	A	Var%	LOD	A	Var%	LOD	A+D	Var%
		E3	SWU11976	SWU11950							4.65	0.03	11.42
	qFE-Chr2-2	E3	TMB1268	JESPR304							4.45	0.03	9.32
	qFE-Chr2-3	E3	PGML0700	SWU12016							4.43	0.04	17.31
	qFE-Chr2-4	E1	SWU12343	SWU14060	3.48	0.04	6.32						
		E1	SWU12343	SWU14060				5.99	0.06	11.63			
	qFE-Chr2-5	E1	MGHES24	ICR11064	5.10	0.05	9.01						
	qFE-Chr5-1	E1	NAU4034	SWU17713							5.26	0.04	10.77
		E1	NAU4034	SWU17713	10.61	0.07	20.41						
		E3	NAU4034	SWU17713	4.13	0.03	8.56						
		E1	NAU4034	SWU17713				5.40	0.06	10.35			
	qFE-Chr5-2	E1	PGML4350	SWU17781							3.04	0.03	6.59
	qFE-Chr6-1	E3	HAU1460	HAU1371				6.14	0.05	12.31			
	qFE-Chr10-1	E3	NAU3404	SWU20501b				3.50	-0.04	6.75			
	qFE-Chr11-1	E1	CER0098	CGR5421							3.24	0.03	8.62
	qFE-Chr18-1	E3	SWU21800	CIR099	3.63	0.04	13.70						
FM	***qFM-Chr2-1***	E3	SWU11887	SWU11976	2.01	-0.07	4.97						
		E2	SWU11887	SWU11976				2.98	-0.08	8.14			
		E2	SWU11887	SWU11976							2.01	-0.06	5.28
		E2	SWU11887	SWU11976	7.50	-0.12	15.50						
		E3	SWU11976	SWU11950				2.72	-0.09	5.82			
	qFM-Chr2-2	E2	PGML0700	SWU12016							2.25	-0.06	6.95
		E2	PGML0700	SWU12016	2.08	-0.07	5.37						
	qFM-Chr2-3	E2	ICR11064	NAU935	3.91	0.08	6.74						
	qFM-Chr4-1	E1	ICR01729	SWU16781	3.66	0.11	9.99						
	qFM-Chr4-2	E1	JESPR295	SWU16782	3.51	0.09	6.98						
	qFM-Chr4-3	E1	SWU16783	NAU3868	2.57	0.11	9.84						
		E3	NAU3868	SWU21617				2.13	0.07	3.76			
	qFM-Chr7-1	E3	HAU1483b	Gh145							3.61	-0.05	6.89
	qFM-Chr8-1	E1	DC20094	HAU1470b				3.02	-0.11	8.88			
	qFM-Chr9-1	E3	SWU15194	HAU190				4.59	0.18	25.93			
		E3	SWU15194	HAU190	3.14	0.10	9.86						
	qFM-Chr14-1	E3	SWU14224	DPL0565							2.78	0.05	7.19
		E3	SWU14224	DPL0565	3.07	0.09	7.59						
	qFM-Chr14-2	E3	ICR12037	CGR5675				3.20	0.10	8.08			
	qFM-Chr14-3	E3	PGML1368	PGML1568				3.05	0.09	6.04			
	qFM-Chr15-1	E3	DC40175	SWU11630							6.40	0.07	12.70
		E3	DC40175	SWU11630	5.28	0.10	9.85						
		E3	DC40175	SWU11630				2.80	0.08	5.26			
	qFM-Chr15-2	E2	CGR6889	DPL0182	5.08	0.09	9.32						
	qFM-Chr19-1	E3	NAU5330	Gh72				2.76	0.09	5.59			
		E1	NAU5330	Gh72							2.53	0.05	5.39
		E1	NAU5330	Gh72	2.97	0.08	6.03						
		E3	NAU5330	Gh72	5.48	0.11	10.49						
		E1	NAU5330	Gh72				2.55	0.08	5.14			

Trait	QTL	Env	Marker interval		RIL′			RIL			BC		
					LOD	A	Var%	LOD	A	Var%	LOD	A+D	Var%
	qFM-Chr19-2	E1	NAU833a	NAU1269							2.82	0.06	5.79
		E1	NAU833a	NAU1269	2.74	0.08	5.43						
		E3	NAU833a	NAU1269	4.12	0.09	7.85						
	qFM-Chr22-1	E2	SWU21586	PGML1712							3.04	0.06	6.42
	qFM-Chr26-1	E1	SWU17432	SWU17395	2.32	-0.08	5.22						
		E3	SWU17432	SWU17395	2.02	-0.07	4.01						
	qFM-Chr26-2	E1	NAU2175	SWU17336							4.42	-0.07	9.90
		E2	NAU2175	SWU17336	3.97	-0.09	8.00						
	qFM-Chr26-3	E1	SWU17336	NAU5072							3.11	-0.06	7.42
	qFM-Chr26-4	E1	SWU16753	SWU16780				3.43	-0.10	6.81			
		E2	SWU16753	SWU16780				2.22	-0.06	4.39			
					**XZV hybrid**								
FL	***qFL-Chr2-1***	E2	SWU12490	DPL0200	2.97	0.38	6.57						
		E3	SWU12490	DPL0200				2.20	0.33	5.56			
	qFL-Chr2-2	E1	TMB1268	SWU11976				2.75	0.31	5.73			
		E3	SWU11976	SWU12001				3.95	0.37	7.17			
		E2	SWU11976	SWU12001				3.07	0.39	6.30			
		E3	SWU11976	SWU12001	4.33	0.35	8.13						
		E3	SWU11976	SWU12001							4.23	0.25	10.20
	qFL-Chr13-1	E2	NAU3398	CGR5331							2.07	0.22	4.58
		E3	NAU3398	CGR5331				2.43	0.28	4.32			
	qFL-Chr14-1	E1	HAU1000	TMB1931							2.67	0.20	5.37
		E3	HAU1000	TMB1931							2.51	0.19	5.72
		E2	HAU1000	TMB1931				2.16	0.37	5.70			
		E3	HAU1000	TMB1931	6.52	0.48	14.93						
		E3	HAU1000	TMB1931				6.73	0.53	15.12			
	qFL-Chr19-1	E1	NAU2894	NAU2893	2.61	0.28	5.18						
		E3	HAU3069	SWU17789	2.56	0.27	4.65						
	qFL-Chr20-1	E1	CER0167	SWU20658							2.03	0.18	4.76
		E1	SWU20658	CGR6154	2.07	0.30	6.30						
	qFL-Chr21-1	E1	BNL1552	CGR5148				3.42	-0.58	19.64			
	qFL-Chr21-2	E1	HAU0423	JESPR154							3.37	-0.25	9.25
	qFL-Chr23-1	E1	NAU3588	NAU5373a	2.57	-0.27	5.05						
		E3	NAU5373a	NAU5373b				2.06	-0.26	3.66			
		E2	NAU5373b	HAU2648				3.69	-0.46	7.67			
	qFL-Chr25-1	E3	HAU3012	SWU19676	3.40	0.44	12.84						
		E1	SWU19676	NAU2968				2.53	0.28	4.76			
FU	*qFU-Chr14-1*	E3	ICR00401	ICR03105							3.24	0.29	10.58
	qFU-Chr23-1	E3	SWU0506	SHIN0272							3.80	0.27	10.43
	qFU-Chr23-2	E1	NAU2140	DC40286				2.28	-0.38	9.89			
		E1	DC40286	PGML1434							3.40	-0.26	7.57
FS	*qFS-Chr14-1*	E1	CIR228	DPL0502				4.33	0.53	8.17			
	qFS-Chr14-2	E2	NAU4045	ICR03943							3.16	0.37	8.08
	qFS-Chr18-1	E1	TMB1638	CGR6812							3.25	-0.40	12.53

Trait	QTL	Env	Marker interval		RIL′			RIL			BC		
					LOD	A	Var%	LOD	A	Var%	LOD	A+D	Var%
	qFS-Chr21-1	E1	BNL1552	CGR5148				3.34	-0.52	7.88			
		E3	BNL1552	CGR5148							2.95	-0.29	12.82
	qFS-Chr21-2	E3	SHIN0337	SWU16370	3.09	-0.28	6.59						
		E1	CGR5748	PGML2500	4.08	-0.47	7.99						
		E3	CGR5748	PGML2500				2.25	-0.25	4.27			
	qFS-Chr23-1	E3	NAU2140	DC40286				2.27	0.32	7.01			
		E1	NAU2140	DC40286							2.23	-0.32	8.27
	qFS-Chr23-2	E2	NAU5373a	NAU5373b				3.70	-0.50	7.30			
	qFS-Chr26-1	E3	HAU1571	CGR6477	4.34	-0.37	11.33						
		E3	HAU1571	CGR6477				5.86	-0.47	14.86			
		E2	CGR6477	PGML2562							2.52	-0.28	6.07
		E1	CGR6477	PGML2562							4.29	-0.37	10.44
		E2	CGR6477	PGML2562	4.41	-0.54	11.10						
		E1	CGR6477	PGML2562				5.92	-0.66	12.93			
		E1	CGR6477	PGML2562	5.79	-0.65	15.03						
FE	*qFE-Chr1-1*	E3	SWU14616	SWU14077	3.56	0.05	14.37						
	qFE-Chr1-2	E1	PGML2498	SWU14490	2.61	0.04	7.81						
		E3	PGML2498	SWU14490				2.43	0.03	5.45			
	qFE-Chr14-1	E1	CIR228	DPL0502				5.07	0.06	10.22			
	qFE-Chr14-2	E1	DPL0502	ICR00401				4.96	0.09	20.58			
	qFE-Chr23-1	E3	SWU0506	SHIN0272				4.35	0.05	12.85			
	qFE-Chr26-1	E1	HAU1571	CGR6477							2.83	-0.04	9.36
		E1	CGR6477	PGML2562	3.86	-0.05	9.89						
		E1	CGR6477	PGML2562				3.52	-0.06	8.57			
FM	*qFM-Chr1-1*	E3	SWU14514	Gh120	3.48	0.10	6.84						
	qFM-Chr1-2	E3	NAU2697	SWU0320	3.32	-0.11	8.38						
	qFM-Chr2-1	E3	SWU12490	DPL0200	4.87	-0.12	10.23						
	qFM-Chr14-1	E2	TMB0071	HAU1000							5.09	-0.08	10.73
		E3	TMB0071	HAU1000	3.73	-0.10	7.25						
		E3	TMB0071	HAU1000				5.07	-0.12	9.91			
		E1	HAU1000	TMB1931							2.32	-0.05	3.97
		E1	HAU1000	TMB1931	3.94	-0.11	8.81						
		E1	HAU1000	TMB1931				5.86	-0.14	14.06			
	qFM-Chr16-1	E1	HAU1129	C2_0011B				2.18	0.09	5.50			
		E1	SWU10211	SWU10266	3.23	0.09	6.06						
		E3	SWU10211	SWU10266				2.12	0.08	4.25			
	qFM-Chr21-1	E3	CGR5748	PGML2500							3.30	0.06	6.45
	qFM-Chr23-1	E3	CGR5158	HAU1758							3.90	-0.08	10.67
	qFM-Chr23-2	E1	MUSB994	NAU2238	4.28	-0.12	11.91						
		E2	MUSB994	NAU2238							2.77	-0.06	6.14
		E2	NAU2238	NAU3588				6.70	-0.13	13.59			
		E2	NAU2238	NAU3588	5.24	-0.11	10.71						
	qFM-Chr23-3	E1	NAU3588	NAU5373a	4.75	-0.11	9.05						
		E1	NAU5373b	HAU2648							6.20	-0.09	11.93

Trait	QTL	Env	Marker interval		RIL′			RIL			BC		
					LOD	A	Var%	LOD	A	Var%	LOD	A+D	Var%
	qFM-Chr25-1	E2	HAU3012	SWU19676				4.17	-0.15	20.63			
	qFM-Chr25-2	E1	SWU19412	NAU3112				3.23	0.12	10.16			
		E3	SWU19412	NAU3112				2.97	0.11	8.58			
	qFM-Chr25-3	E3	HAU1355	BNL3098							2.39	0.05	4.62
		E1	HAU1355	BNL3098				3.55	0.09	6.59			
	qFM-Chr26-1	E1	CGR6477	PGML2562							3.81	-0.07	8.73
		E2	PGML2562	HAU1371	3.39	-0.08	6.38						

Bold figures indicate the QTL was detected in more than two environments or populations simultaneously

QTL identified from Cartographer and the M-QTL from ICIMapping were compared, and common QTLs are shown as blue figures

Env., Environment, E1: Handan; E2: Cangzhou; E3: Xiangyang

Effect, the genetic expectation of a QTL effect obtained is the additive effect (A) when estimated from the RILs and RIL′s, the additive and dominance effects (A+D) from the BC mean values

Var%, Phenotypic variation explained by a single QTL
