# Supplementary material for: Genetic Analysis and QTL Detection on Fiber Traits Using Two Recombinant Inbred Lines and Their Backcross Populations in Upland Cotton
Source: G3 (Bethesda). 2016 Jun 23;6(9):2717–24. doi: 10.1534/g3.116.031302 (PMC5015930; doi:10.1534/g3.116.031302)
Supplement: Supplemental Material [file supp_g3.116.031302_TableS3.pdf]

**Table S3** Main effects and environmental interactions detected for fiber quality traits in RIL and RILV populations by ICIMapping 4.0

| Trait | Chr. | Position (cM) | Flanking markers | | LOD | V(A) | V(AE) | A | AE1 | AE2 | AE3 |
|---|---|---|---|---|---|---|---|---|---|---|---|
| RIL population | | | | | | | | | | | |
| FL | 2 | 7 | DPL0217 | SWU12025 | 4.68 | 2.09 | 0.39 | 0.15 | 0.03 | -0.09 | 0.05 |
| | 2 | 66 | PGML0700 | SWU12016 | 5.22 | 3.32 | 0.11 | 0.19 | -0.01 | 0.05 | -0.03 |
| | 4 | 85 | SWU16783 | NAU3868 | 3.16 | 1.70 | 0.07 | -0.13 | -0.04 | 0.02 | 0.02 |
| | 5 | 23 | SWU20917 | NAU6240 | 7.04 | 4.63 | 0.29 | 0.22 | -0.03 | 0.08 | -0.04 |
| | 5 | 114 | SWU17715 | Gh388 | 7.89 | 3.64 | 2.02 | 0.19 | 0.12 | 0.08 | -0.20 |
| | 5 | 121 | PGML4457 | MUSS193 | 5.51 | 1.36 | 1.48 | 0.12 | -0.07 | -0.11 | 0.17 |
| | 10 | 42 | SWU20260 | Gh144 | 6.27 | 4.11 | 1.01 | -0.21 | 0.04 | -0.14 | 0.10 |
| | 10 | 44 | Gh320 | HAU0635 | 3.48 | 1.34 | 0.48 | -0.12 | -0.08 | 0.09 | -0.01 |
| | 18 | 117 | NAU748 | SWU22192 | 4.47 | 2.56 | 0.51 | -0.16 | -0.06 | -0.04 | 0.10 |
| | 19 | 120 | SWU14431b | SWU17782 | 4.21 | 2.62 | 0.02 | 0.16 | 0.00 | 0.02 | -0.02 |
| | 21 | 168 | DPL0050a | BNL3171 | 4.85 | 2.61 | 0.56 | -0.16 | -0.09 | 0.00 | 0.09 |
| | 24 | 29 | PGML4657 | Gh454 | 3.69 | 2.11 | 0.05 | -0.15 | -0.03 | 0.02 | 0.01 |
| FU | 1 | 161 | CGR5663 | NAU2343 | 2.90 | 0.44 | 1.61 | 0.06 | 0.14 | -0.15 | 0.02 |
| | 2 | 3 | SWU12126 | SWU12147 | 2.51 | 0.63 | 2.10 | 0.07 | -0.07 | 0.19 | -0.12 |
| | 3 | 2 | HAU2424 | CER0028 | 2.75 | 1.86 | 0.74 | -0.13 | 0.04 | -0.11 | 0.07 |
| | 6 | 87 | CGR5801 | SWU19249 | 2.78 | 2.04 | 0.07 | 0.13 | 0.02 | 0.01 | -0.03 |
| | 13 | 45 | SWU13032 | DPL0308 | 2.51 | 1.05 | 0.70 | -0.10 | -0.10 | 0.01 | 0.09 |
| | 13 | 69 | NAU2893 | Gh157 | 2.67 | 1.98 | 0.40 | -0.13 | 0.02 | -0.08 | 0.06 |
| | 25 | 116 | SWU19144 | Gh220 | 2.59 | 0.14 | 2.33 | -0.03 | 0.15 | -0.19 | 0.04 |
| | 28 | 84 | TMB2386 | SWU12343 | 2.89 | 0.35 | 1.63 | 0.05 | -0.07 | -0.09 | 0.17 |
| FS | 1 | 41 | ICR03725 | SWU10987 | 4.77 | 2.23 | 1.52 | -0.18 | -0.20 | 0.15 | 0.06 |
| | 1 | 159 | CGR5663 | NAU2343 | 3.01 | 1.66 | 0.39 | 0.16 | -0.01 | 0.10 | -0.09 |
| | 2 | 65 | SWU11950 | TMB1268 | 2.61 | 1.70 | 0.07 | 0.16 | 0.02 | 0.02 | -0.05 |
| | 3 | 1 | HAU2424 | CER0028 | 3.20 | 1.96 | 0.09 | -0.17 | -0.05 | 0.03 | 0.02 |
| | 4 | 3 | NAU2701 | SWU18876 | 3.14 | 1.39 | 1.29 | 0.14 | 0.19 | -0.07 | -0.12 |
| | 5 | 30 | SWU20917 | NAU6240 | 12.51 | 8.53 | 0.33 | 0.36 | 0.10 | -0.04 | -0.06 |
| | 5 | 120 | HAU1603 | PGML4457 | 3.14 | 1.71 | 0.23 | 0.16 | 0.07 | -0.07 | 0.00 |
| | 8 | 33 | DC20094 | HAU1470b | 2.52 | 1.38 | 0.40 | 0.14 | 0.11 | -0.07 | -0.04 |
| | 13 | 11 | CGR5554 | SHIN1462 | 3.74 | 2.24 | 0.21 | -0.18 | -0.08 | 0.06 | 0.02 |
| | 13 | 29 | NAU3468 | SWU22309 | 3.51 | 2.26 | 0.26 | -0.19 | -0.05 | -0.04 | 0.09 |
| | 13 | 48 | DPL0308 | DPL0535 | 3.29 | 1.72 | 0.48 | -0.16 | 0.01 | -0.11 | 0.10 |
| | 13 | 55 | PGML0014 | CGR6732 | 2.78 | 1.43 | 0.41 | -0.15 | 0.04 | -0.11 | 0.07 |
| | 14 | 58 | NAU3308 | HAU1057 | 5.02 | 2.64 | 0.26 | -0.20 | -0.04 | 0.09 | -0.05 |
| | 18 | 78 | CIR099 | NAU748 | 6.12 | 3.67 | 1.01 | 0.23 | 0.17 | -0.09 | -0.08 |
| | 18 | 139 | DC40150 | ICR02849 | 3.29 | 1.76 | 0.26 | -0.16 | 0.02 | -0.08 | 0.07 |

| Trait | Chr. | Position (cM) | Flanking markers | | LOD | V(A) | V(AE) | A | AE1 | AE2 | AE3 |
|---|---|---|---|---|---|---|---|---|---|---|---|
| | 19 | 35 | NAU3437 | NAU2894 | 6.97 | 3.65 | 0.40 | 0.23 | 0.05 | -0.11 | 0.06 |
| | 21 | 70 | SWU16488 | SWU16138 | 4.30 | 2.67 | 0.03 | -0.20 | -0.02 | -0.01 | 0.03 |
| | 21 | 146 | JESPR154 | SWU14431a | 4.86 | 2.75 | 0.43 | -0.21 | 0.07 | -0.11 | 0.04 |
| | 21 | 171 | BNL3171 | CGR5808 | 3.42 | 2.04 | 0.12 | -0.17 | -0.05 | 0.05 | 0.01 |
| | 25 | 25 | SWU19815 | BNL3594 | 2.52 | 1.48 | 0.43 | 0.15 | 0.11 | -0.04 | -0.07 |
| | 26 | 26 | SWU17395 | DC30107 | 2.78 | 1.80 | 0.08 | -0.16 | -0.04 | -0.01 | 0.05 |
| FE | 2 | 4 | SWU12147 | CGR6695 | 3.62 | 3.53 | 0.11 | 0.03 | 0.01 | -0.01 | - |
| | 5 | 114 | SWU17715 | Gh388 | 4.20 | 2.31 | 3.24 | 0.02 | 0.03 | -0.03 | - |
| | 5 | 120 | HAU1603 | PGML4457 | 2.84 | 1.19 | 0.94 | 0.02 | -0.02 | 0.02 | - |
| | 6 | 0 | ICR00143 | CGR5108 | 2.70 | 0.47 | 1.62 | -0.01 | 0.02 | -0.02 | - |
| | 6 | 97 | CGR5124 | HAU1460 | 6.62 | 4.69 | 0.67 | 0.03 | -0.01 | 0.01 | - |
| | 10 | 8 | NAU3404 | SWU20501b | 3.55 | 2.56 | 0.34 | -0.03 | 0.01 | -0.01 | - |
| | 24 | 31 | PGML4657 | Gh454 | 3.56 | 3.56 | 0.56 | -0.03 | -0.01 | 0.01 | - |
| | 28 | 85 | SWU12343 | SWU14060 | 4.74 | 4.13 | 1.90 | 0.03 | 0.02 | -0.02 | - |
| FM | 2 | 56 | SWU11887 | SWU11976 | 7.52 | 4.81 | 0.11 | -0.07 | 0.01 | -0.01 | 0.00 |
| | 4 | 80 | SWU16783 | NAU3868 | 3.72 | 2.58 | 0.33 | 0.05 | 0.01 | -0.03 | 0.02 |
| | 5 | 131 | NBRI0694 | DPL0022 | 3.06 | 2.16 | 0.38 | -0.05 | -0.01 | 0.03 | -0.02 |
| | 7 | 28 | SWU10064 | NAU3181 | 3.58 | 2.19 | 0.22 | -0.05 | 0.01 | 0.01 | -0.02 |
| | 8 | 36 | DC20094 | HAU1470b | 4.58 | 3.17 | 0.20 | -0.06 | -0.02 | 0.01 | 0.01 |
| | 9 | 57 | SWU15194 | HAU190 | 4.23 | 2.41 | 1.04 | 0.05 | 0.00 | -0.04 | 0.04 |
| | 14 | 106 | PGML1568 | Gh529 | 3.06 | 2.10 | 0.21 | 0.05 | 0.00 | -0.02 | 0.02 |
| | 15 | 1 | DC40175 | SWU11630 | 4.79 | 3.46 | 0.14 | 0.06 | 0.01 | -0.02 | 0.01 |
| | 18 | 1 | SWU22287 | SWU22290 | 2.72 | 1.98 | 0.18 | 0.05 | 0.01 | -0.02 | 0.01 |
| | 19 | 6 | NAU5330 | Gh72 | 5.33 | 3.89 | 0.29 | 0.07 | 0.01 | -0.03 | 0.01 |
| | 19 | 11 | DC40122 | NAU833a | 3.89 | 2.69 | 0.60 | 0.06 | 0.02 | -0.04 | 0.02 |
| | 19 | 188 | TMB0107 | NAU3217 | 3.23 | 2.13 | 0.46 | -0.05 | -0.01 | 0.03 | -0.02 |
| | 20 | 22 | CGR6154 | SWU20246 | 4.70 | 3.25 | 0.30 | -0.06 | 0.01 | 0.01 | -0.03 |
| | 26 | 70 | DPL0491 | Gh64 | 3.61 | 1.84 | 0.45 | -0.05 | -0.01 | -0.02 | 0.03 |
| | 27 | 57 | CGR6356 | SWU11632 | 3.08 | 2.00 | 0.22 | -0.05 | 0.01 | 0.01 | -0.02 |
| | 31 | 36 | SWU16753 | SWU16780 | 5.47 | 3.99 | 0.61 | -0.07 | -0.04 | 0.02 | 0.02 |
| RILV population | | | | | | | | | | | |
| FL | 2 | 28 | SWU12490 | DPL0200 | 3.43 | 2.44 | 0.02 | 0.22 | -0.01 | -0.02 | 0.03 |
| | 2 | 114 | SWU11976 | SWU12001 | 8.20 | 6.16 | 0.07 | 0.35 | -0.05 | 0.03 | 0.03 |
| | 3 | 28 | SWU12841 | SWU12838b | 3.85 | 2.79 | 0.11 | 0.24 | -0.04 | 0.06 | -0.02 |
| | 6 | 112 | DPL0590 | NAU2971 | 2.72 | 1.65 | 0.17 | 0.18 | 0.04 | -0.08 | 0.04 |
| | 12 | 50 | SWU16858 | TMB0327 | 2.51 | 1.60 | 0.08 | -0.18 | -0.05 | 0.05 | 0.00 |
| | 13 | 33 | NAU3398 | CGR5331 | 5.21 | 3.63 | 0.00 | 0.27 | 0.00 | -0.01 | 0.01 |
| | 19 | 17 | NAU2893 | HAU3069 | 3.91 | 2.78 | 0.00 | 0.23 | 0.00 | 0.01 | -0.01 |

| Trait | Chr. | Position (cM) | Flanking markers | | LOD | V(A) | V(AE) | A | AE1 | AE2 | AE3 |
|---|---|---|---|---|---|---|---|---|---|---|---|
| | 19 | 44 | SWU17789 | SWU17882 | 2.87 | 2.12 | 0.05 | 0.20 | -0.04 | 0.04 | 0.00 |
| | 20 | 29 | HAU1314 | SWU20035 | 3.40 | 2.72 | 0.05 | 0.23 | -0.03 | 0.04 | -0.01 |
| | 21 | 4 | BNL1552 | CGR5148 | 3.76 | 2.40 | 0.20 | -0.22 | -0.06 | 0.09 | -0.02 |
| | 21 | 65 | CGR5748 | PGML2500 | 3.07 | 2.20 | 0.07 | -0.21 | -0.02 | -0.03 | 0.05 |
| | 23 | 282 | NAU5373b | HAU2648 | 5.68 | 4.02 | 0.59 | -0.28 | 0.12 | -0.14 | 0.02 |
| | 25 | 22 | HAU3012 | SWU19676 | 3.96 | 2.23 | 0.42 | 0.21 | 0.06 | -0.13 | 0.07 |
| | 32 | 7 | HAU1000 | TMB1931 | 10.54 | 7.54 | 0.26 | 0.39 | 0.00 | -0.09 | 0.09 |
| | 33 | 10 | BNL3661 | PGML4891 | 4.26 | 3.09 | 0.05 | -0.25 | 0.03 | -0.05 | 0.02 |
| | 37 | 24 | HAU0423 | JESPR154 | 2.65 | 1.85 | 0.09 | -0.19 | -0.05 | -0.01 | 0.05 |
| | 38 | 23 | NAU2450 | PGML1942 | 2.56 | 1.83 | 0.12 | 0.19 | -0.06 | 0.06 | -0.01 |
| | 39 | 76 | SWU16437 | SWU16432 | 2.69 | 1.93 | 0.03 | 0.20 | -0.03 | 0.02 | 0.02 |
| FS | 1 | 36 | SWU17434 | SWU14616 | 3.09 | 1.63 | 0.59 | -0.21 | -0.17 | 0.11 | 0.06 |
| | 5 | 27 | PGML0120 | SWU20917 | 2.52 | 1.19 | 0.10 | -0.18 | 0.03 | 0.04 | -0.07 |
| | 12 | 56 | ICR03107 | HAU3373 | 4.16 | 2.79 | 0.08 | -0.27 | 0.00 | -0.05 | 0.06 |
| | 14 | 5 | HAU0883 | CIR228 | 3.15 | 1.44 | 1.24 | 0.20 | 0.24 | -0.04 | -0.20 |
| | 21 | 3 | BNL1552 | CGR5148 | 5.86 | 4.06 | 0.30 | -0.33 | -0.12 | 0.02 | 0.10 |
| | 21 | 65 | CGR5748 | PGML2500 | 5.03 | 3.47 | 0.10 | -0.30 | 0.00 | -0.06 | 0.06 |
| | 23 | 280 | NAU3588 | NAU5373a | 5.02 | 3.53 | 0.86 | -0.31 | 0.06 | -0.21 | 0.15 |
| | 26 | 27 | CGR6477 | PGML2562 | 10.14 | 6.91 | 0.29 | -0.43 | -0.12 | 0.07 | 0.06 |
| | 33 | 10 | BNL3661 | PGML4891 | 5.64 | 3.75 | 0.20 | -0.32 | 0.05 | -0.10 | 0.05 |
| FE | 1 | 78 | PGML2498 | SWU14490 | 2.53 | 2.37 | 0.01 | 0.03 | 0.00 | - | 0.00 |
| | 2 | 120 | SWU11976 | SWU12001 | 2.87 | 1.78 | 0.43 | 0.02 | -0.01 | - | 0.01 |
| | 14 | 6 | CIR228 | DPL0502 | 2.62 | 1.97 | 2.37 | 0.02 | 0.03 | - | -0.03 |
| | 15 | 8 | NAU3736 | SWU11691 | 2.54 | 2.86 | 0.56 | -0.03 | -0.01 | - | 0.01 |
| | 23 | 154 | SWU0506 | SHIN0272 | 3.39 | 0.96 | 1.46 | 0.02 | -0.02 | - | 0.02 |
| | 26 | 27 | CGR6477 | PGML2562 | 2.70 | 3.07 | 0.59 | -0.03 | -0.01 | - | 0.01 |
| FM | 1 | 11 | Gh529 | SWU17434 | 3.43 | 2.45 | 0.01 | 0.06 | 0.00 | 0.00 | 0.00 |
| | 1 | 320 | NAU0748 | NAU2697 | 2.94 | 2.02 | 0.01 | -0.05 | 0.00 | 0.00 | 0.00 |
| | 2 | 21 | DPL0041 | SWU12490 | 5.04 | 3.68 | 0.09 | -0.07 | 0.00 | 0.01 | -0.01 |
| | 2 | 114 | SWU11976 | SWU12001 | 2.78 | 1.50 | 0.55 | -0.04 | -0.01 | 0.04 | -0.02 |
| | 3 | 0 | SWU12840 | NAU2742 | 4.35 | 2.90 | 0.07 | -0.06 | -0.01 | 0.01 | 0.01 |
| | 3 | 28 | SWU12841 | SWU12838b | 4.85 | 3.29 | 0.11 | -0.07 | -0.01 | 0.02 | -0.01 |
| | 6 | 82 | HAU2768 | HAU0483 | 2.55 | 1.40 | 0.31 | 0.04 | -0.03 | 0.02 | 0.01 |
| | 8 | 19 | HAU0810 | TMB2904 | 3.09 | 2.17 | 0.03 | 0.05 | 0.00 | -0.01 | 0.01 |
| | 10 | 92 | SWU19932 | HAU0635 | 2.86 | 1.89 | 0.07 | 0.05 | -0.01 | 0.01 | 0.00 |
| | 12 | 123 | SWU17197 | Gh631 | 2.98 | 2.03 | 0.11 | -0.05 | -0.01 | 0.02 | -0.01 |
| | 14 | 104 | NAU4045 | ICR03943 | 3.21 | 2.09 | 0.16 | 0.05 | 0.02 | -0.02 | 0.00 |
| | 15 | 0 | NAU3736 | SWU11691 | 3.24 | 2.19 | 0.17 | -0.05 | 0.01 | 0.01 | -0.02 |

| Trait | Chr. | Position (cM) | Flanking markers | | LOD | V(A) | V(AE) | A | AE1 | AE2 | AE3 |
|---|---|---|---|---|---|---|---|---|---|---|---|
| | 16 | 45 | HAU1129 | C2_0011B | 3.93 | 2.81 | 0.10 | 0.06 | 0.00 | -0.01 | 0.01 |
| | 17 | 38 | HAU1413 | CGR5576 | 3.12 | 2.11 | 0.03 | -0.05 | -0.01 | 0.00 | 0.01 |
| | 21 | 175 | SWU16408 | BNL3171 | 4.71 | 3.05 | 0.34 | -0.06 | -0.01 | 0.03 | -0.02 |
| | 23 | 272 | MUSB994 | NAU2238 | 5.20 | 2.91 | 0.57 | -0.06 | 0.01 | -0.04 | 0.03 |
| | 24 | 83 | SWU13121 | CGR6079 | 5.08 | 3.02 | 0.79 | -0.06 | -0.04 | 0.04 | 0.00 |
| | 25 | 21 | HAU3012 | SWU19676 | 3.50 | 2.17 | 0.12 | -0.05 | 0.00 | -0.01 | 0.02 |
| | 25 | 96 | HAU1355 | BNL3098 | 4.23 | 1.79 | 1.18 | 0.05 | 0.05 | -0.02 | -0.04 |
| | 26 | 53 | DC30107 | DPL0070 | 3.57 | 2.24 | 0.13 | -0.05 | -0.02 | 0.00 | 0.02 |
| | 28 | 0 | BNL3545 | PGML3983 | 3.18 | 2.14 | 0.08 | 0.05 | -0.01 | 0.01 | 0.01 |
| | 32 | 2 | TMB0071 | HAU1000 | 10.78 | 7.90 | 0.47 | -0.10 | -0.01 | 0.03 | -0.02 |
| | 33 | 8 | BNL3661 | PGML4891 | 3.78 | 2.29 | 0.21 | 0.05 | -0.01 | 0.02 | -0.01 |

Chr represents the linkage group number of the loci being tested in the analysis.

V(A): Phenotypic variation explained by additive effect at the current scanning position.

V(AE): Phenotypic variation explained by additive by environment effect at the current scanning position.

A, the additive effect.
