# Supplementary material for: Genetic Analysis and QTL Detection on Fiber Traits Using Two Recombinant Inbred Lines and Their Backcross Populations in Upland Cotton
Source: G3 (Bethesda). 2016 Jun 23;6(9):2717–24. doi: 10.1534/g3.116.031302 (PMC5015930; doi:10.1534/g3.116.031302)
Supplement: Supplemental Material [file supp_g3.116.031302_TableS4.pdf]

Table S4 Main effects and environmental interactions detected for fiber quality traits in BCF_1 and $BCVF_1$ populations by ICIMapping 4.0

Trait	Chr.	Position (cM)	Flanking markers		LOD	V(A)	V(AE)	A	AE1	AE2	AE3
BCF_1 population											
FL	2	10	SWU12025	SWU11889	3.83	2.34	0.63	0.11	-0.07	0.07	0.00
	5	54	NAU6240	PGML1671	4.68	2.36	0.36	0.11	-0.05	-0.01	0.06
	5	114	SWU17715	Gh388	4.76	1.82	1.27	0.10	0.11	-0.02	-0.09
	5	129	SWU17781	CGR5025	4.02	1.36	0.68	0.08	-0.05	-0.03	0.08
	10	23	SWU20501b	CGR5873	3.56	2.45	0.12	-0.11	-0.01	-0.02	0.04
	17	87	CGR5871	SWU12876	2.97	1.96	1.49	0.10	-0.07	0.12	-0.05
	21	21	SWU16649	BNL1552	2.71	1.91	0.98	-0.10	0.04	-0.10	0.06
	21	157	SWU15915	SWU0189	3.83	2.81	0.38	-0.12	0.03	-0.06	0.03
	27	71	CGR5056	DC40052	3.30	2.50	0.57	-0.12	0.03	-0.08	0.05
FU	1	23	SWU10930	ICR03295	3.58	2.08	0.56	-0.13	-0.09	0.06	0.03
	7	41	NAU3181	SHIN0376	2.59	1.54	0.18	0.11	0.05	-0.04	-0.01
	10	42	SWU20260	Gh144	3.52	1.07	2.53	-0.09	0.16	-0.19	0.03
	14	76	SWU14543	ICR12037	2.81	0.54	1.97	-0.06	0.02	-0.16	0.14
	17	40	CGR5576	NAU3765	2.89	1.21	1.48	0.10	0.04	0.11	-0.15
	20	0	SWU20700	CGR5548	2.81	0.56	1.10	0.07	0.07	-0.13	0.07
	22	104	PGML0695	SWU20813	3.29	1.70	0.30	-0.12	-0.05	0.07	-0.02
	26	148	C2_0135	PGML2321	3.44	2.08	0.82	-0.13	-0.11	0.04	0.07
FS	1	99	NAU2218	SWU11191	4.30	1.82	0.93	-0.12	0.12	-0.06	-0.06
	1	126	NAU3384	CGR5663	6.01	2.30	0.81	0.13	-0.02	-0.08	0.11
	5	53	NAU6240	PGML1671	4.25	3.07	0.31	0.15	0.05	0.01	-0.06
	5	114	SWU17715	Gh388	3.76	1.42	1.24	0.10	0.10	-0.13	0.03
	21	171	BNL3171	CGR5808	4.68	3.25	0.90	-0.16	-0.10	-0.01	0.10
	21	253	DPL0777	CGR5217	2.90	1.53	0.50	-0.11	0.08	-0.07	-0.01
	26	70	DPL0491	Gh64	3.43	2.04	0.62	0.13	0.10	-0.06	-0.03
FE	2	59	SWU11976	SWU11950	4.19	3.27	0.56	0.02	-0.01	0.01	-
	11	71	CER0098	CGR5421	3.07	3.07	1.16	0.02	0.01	-0.01	-
FM	1	41	ICR03725	SWU10987	2.98	2.18	0.28	0.03	0.02	0.00	-0.01
	7	87	SWU10205	HAU1483a	3.14	0.15	1.62	-0.01	0.01	0.03	-0.04
	14	57	NAU874	SWU13824	2.93	0.23	1.35	0.01	-0.02	-0.02	0.04
	15	1	DC40175	SWU11630	6.42	3.04	0.80	0.04	-0.02	-0.01	0.03
	22	19	SWU21586	PGML1712	2.92	1.14	1.47	0.02	0.01	0.03	-0.04
	26	40	SWU17336	NAU5072	3.89	2.94	0.11	-0.04	-0.01	0.00	0.01
	26	73	Gh64	SWU17257	4.08	2.94	0.42	-0.04	-0.02	0.00	0.02
$BCVF_1$ population											
FL	1	75	PGML2498	SWU14490	2.98	2.06	0.25	0.13	-0.04	0.06	-0.01

Trait	Chr.	Position (cM)	Flanking markers		LOD	V(A)	V(AE)	A	AE1	AE2	AE3
	2	116	SWU11976	SWU12001	4.61	2.32	0.54	0.13	-0.06	-0.03	0.09
	13	33	NAU3398	CGR5331	3.13	2.33	0.17	0.13	-0.05	0.04	0.00
	21	65	CGR5748	PGML2500	3.32	1.94	0.99	-0.12	-0.03	-0.09	0.12
	23	148	ICR06429	SWU0506	2.90	0.01	1.87	-0.01	-0.07	-0.12	0.19
	32	3	TMB0071	HAU1000	5.74	3.96	0.19	0.18	0.05	-0.03	-0.02
	37	23	HAU0423	JESPR154	3.81	2.66	0.12	-0.14	-0.03	-0.01	0.04
FU	8	8	Gh197	HAU0810	2.65	0.56	1.45	0.07	0.14	-0.02	-0.12
	14	39	ICR00401	ICR03105	4.07	0.54	1.41	0.08	-0.12	-0.07	0.19
	23	162	SWU0506	SHIN0272	2.63	0.39	0.93	0.06	-0.13	0.03	0.10
	23	201	DC40286	PGML1434	4.55	0.00	3.63	-0.01	-0.25	0.14	0.11
	23	277	NAU2238	NAU3588	2.65	0.00	1.94	0.00	-0.18	0.10	0.08
	26	20	HAU1571	CGR6477	2.82	0.34	1.07	-0.05	-0.04	0.13	-0.09
FS	1	260	SWU11632	SWU21958	3.25	1.90	0.09	0.14	0.02	-0.04	0.02
	14	96	NAU4045	ICR03943	3.70	1.43	1.59	0.14	-0.06	0.20	-0.14
	21	1	BNL1552	CGR5148	6.13	3.81	0.10	-0.20	0.04	-0.01	-0.03
	26	27	CGR6477	PGML2562	4.82	3.58	0.42	-0.19	-0.03	-0.06	0.09
	32	10	HAU1000	TMB1931	4.74	3.42	0.62	0.19	0.08	0.03	-0.11
FM	2	24	DPL0041	SWU12490	2.63	1.64	0.11	-0.03	0.00	0.01	-0.01
	10	4	NAU3395	CAU0234	2.71	0.01	1.56	0.00	-0.04	0.01	0.03
	15	0	NAU3736	SWU11691	2.95	1.60	0.34	-0.03	0.02	0.00	-0.02
	16	104	SWU18366	SWU18579	2.61	1.57	0.05	0.03	0.01	0.00	0.00
	21	65	CGR5748	PGML2500	4.02	2.39	0.27	0.04	0.01	-0.02	0.01
	23	1	CGR5158	HAU1758	2.89	0.81	1.11	-0.02	0.02	0.01	-0.04
	23	272	MUSB994	NAU2238	3.27	1.15	1.08	-0.03	0.02	-0.03	0.02
	23	282	NAU5373b	HAU2648	5.67	1.16	3.02	-0.03	-0.06	0.03	0.03
	24	83	SWU13121	CGR6079	3.22	1.71	0.37	-0.03	0.02	-0.01	-0.01
	25	96	HAU1355	BNL3098	3.57	1.61	0.87	0.03	-0.01	-0.02	0.03
	26	51	CGR6477	PGML2562	5.18	2.95	0.69	-0.04	-0.02	0.02	0.00
	28	0	BNL3545	PGML3983	4.25	2.36	0.58	0.04	-0.02	0.02	-0.01
	32	2	TMB0071	HAU1000	7.32	4.63	0.27	-0.05	0.01	-0.02	0.00
	33	6	BNL3661	PGML4891	3.10	1.31	0.61	0.03	0.01	-0.02	0.02

See footnotes of supplementary table S3 for explanations
