# Supplementary material for: Genetic Analysis and QTL Detection on Fiber Traits Using Two Recombinant Inbred Lines and Their Backcross Populations in Upland Cotton
Source: G3 (Bethesda). 2016 Jun 23;6(9):2717–24. doi: 10.1534/g3.116.031302 (PMC5015930; doi:10.1534/g3.116.031302)
Supplement: Supplemental Material [file supp_g3.116.031302_TableS5.pdf]

Table S5 Epistatic effects and environmental interactions detected for fiber quality traits in RIL and RILV populations using two-locus analysis by ICIMapping 4.0

Trait	Chi	Flanking markers		Chj	Flanking markers		LOD	V(AA)	V(AAE)	AA	AAE1	AAE2	AAE3
RIL population													
FL	1	NAU3177	ICR03724	2	SWU12393	SWU11013	5.35	2.84	0.54	-0.17	0.06	-0.11	0.05
	1	NAU3177	ICR03724	3	SWU12840	NAU2742	6.47	3.94	0.14	-0.20	0.05	-0.05	0.00
	3	SWU12819	SWU12765	4	SWU18876	SWU12672	5.59	3.20	0.33	-0.18	0.04	-0.08	0.04
	3	SWU12840	NAU2742	6	ICR00143	CGR5108	5.59	3.26	0.23	-0.18	-0.06	0.00	0.06
	4	ICR01729	SWU16781	6	ICR00143	CGR5108	6.02	3.30	0.12	0.19	-0.01	0.05	-0.04
	1	ICR03724	ICR03725	7	SWU10205	HAU1483a	5.33	2.38	0.91	-0.16	0.07	-0.13	0.07
	7	SWU10064	NAU3181	9	Gh27	SWU15194	5.11	2.57	0.60	0.16	-0.05	0.11	-0.06
	6	CGR5801	SWU19249	12	NAU943	DPL0303	5.45	3.24	0.10	0.18	0.03	0.01	-0.04
	5	CGR5025	NBRI0694	14	ICR12037	CGR5675	6.25	3.49	0.45	-0.19	0.06	-0.09	0.03
	11	NAU1014	ICR10344	15	DPL0182	SWU11691	8.29	4.98	0.06	-0.23	-0.03	0.00	0.03
	13	SHIN1462	SWU22374	18	Gh60	SWU22281	6.07	3.52	0.20	0.19	-0.05	0.06	0.00
	9	Gh27	SWU15194	20	CER0167	SWU20064	6.66	4.01	0.05	0.20	-0.02	0.03	-0.01
	1	NAU3384	CGR5663	23	PGML4186	NAU3100	5.67	3.13	0.00	-0.19	-0.01	0.00	0.02
	4	ICR01729	SWU16781	23	PGML4186	NAU3100	5.05	2.19	0.66	0.15	-0.06	0.12	-0.06
	9	NAU5474	Gh158	24	SWU13758	CGR5423	6.54	3.39	0.36	-0.19	0.03	-0.09	0.05
	5	**SWU20917**	**NAU6240**	25	CGR6864	SWU19815	5.55	3.14	0.39	-0.18	0.06	-0.09	0.03
	18	CIR099	NAU748	26	SWU17432	SWU17395	9.86	5.53	0.16	0.24	0.03	0.03	-0.06
	19	NAU5330	Gh72	26	SWU17395	DC30107	5.40	2.85	0.38	0.17	-0.05	0.09	-0.04
	16	SWU10266	DC40065	29	DC20127	DPL0252	5.87	3.21	0.35	0.18	-0.07	0.08	-0.01
	20	CGR6154	SWU20246	29	BNL3261	CGR5111	6.15	3.50	0.32	-0.19	0.07	-0.07	0.00
FU	3	SWU12783	SWU12819	3	SWU12840	NAU2742	6.64	2.49	1.61	0.16	-0.08	-0.10	0.18
	5	PGML1917	SWU17715	9	SWU15157	SWU14934	5.01	1.72	2.19	0.12	-0.14	0.19	-0.06
	11	NAU3695	DPL0050b	17	CGR5576	NAU3765	5.08	2.49	1.21	0.15	-0.10	0.15	-0.05
	3	SWU12732	SWU12783	24	HAU2504	SWU13736	5.19	1.80	2.13	0.13	-0.11	0.18	-0.07
	22	SWU21533	DPL0562	24	SWU13758	CGR5423	5.50	2.05	1.67	-0.14	0.08	-0.17	0.10
	26	NAU2175	SWU17336	26	BNL2495	DPL0491	5.61	3.10	0.35	0.22	-0.04	-0.02	0.06
FS	1	ICR03724	ICR03725	3	SWU12765	NAU3839	6.55	2.82	0.44	-0.21	-0.05	-0.06	0.12
	9	SWU15157	SWU14934	11	NAU3390	NAU2460	6.14	2.94	0.59	-0.21	-0.04	-0.09	0.13
	5	SWU20913	Gh260	16	SWU10266	DC40065	7.11	2.33	1.21	0.19	0.10	0.09	-0.19
	13	SWU22309	SWU22324	17	SWU14627	CGR5871	5.66	3.06	0.10	0.22	0.03	0.03	-0.07
	14	BNL3661	PGML2498	19	NAU5330	Gh72	5.87	2.20	0.72	0.18	0.13	-0.01	-0.12
	4	BNL1167	SWU21415	20	SWU20636	CGR6154	5.57	3.31	0.02	0.23	0.03	-0.01	-0.02
	19	**NAU3437**	**NAU2894**	23	SWU14807	PGML4185	5.00	2.85	0.13	-0.21	-0.01	-0.05	0.06
	11	SWU15972	TMB0628	25	DPL0282	SWU19763	6.12	3.22	0.14	-0.22	-0.03	-0.03	0.07
	17	ICR03391	SWU12838	25	DPL0282	SWU19763	6.43	3.33	0.39	-0.22	-0.10	0.03	0.08
	18	SWU21800	CIR099	26	SWU18488	SWU18672	6.14	3.29	0.20	0.22	0.06	0.01	-0.07
	8	**DC20094**	**HAU1470b**	28	NBRI0014	SWU12107	5.54	2.79	0.17	0.20	0.01	0.05	-0.07
	2	SWU11887	SWU11976	29	DC20127	DPL0252	6.14	3.36	0.03	-0.22	-0.03	0.01	0.02
	16	DPL0048	SWU10266	29	DC20127	DPL0252	5.96	3.14	0.20	0.22	-0.02	0.07	-0.05
	13	NAU2893	Gh157	29	C2_0115	ICR03107	5.29	3.03	0.07	-0.21	-0.05	0.03	0.01
FE	1	NAU3384	CGR5663	13	HAU2558	NAU2893	5.26	4.65	0.48	0.03	0.01	-0.01	-
	8	CGR6508	Gh197	14	SWU14224	DPL0565	5.25	3.34	1.42	-0.03	-0.02	0.02	-
	8	DC20094	HAU1470b	16	SWU10062	SWU10094	6.32	5.84	0.15	-0.04	-0.01	0.01	-
	14	ICR12037	CGR5675	20	SWU20700	CGR5548	5.43	4.08	0.57	0.03	0.02	-0.02	-

Trait	Chi	Flanking markers		Chj	Flanking markers		LOD	V(AA)	V(AAE)	AA	AAE1	AAE2	AAE3
	19	SWU17782	DPL0056	25	CGR6864	SWU19815	5.02	4.76	0.09	0.03	0.01	-0.01	-
	26	SWU17251	C2_0135	28	**SWU12343**	**SWU14060**	5.09	5.24	0.03	0.04	0.00	0.00	-
FM	2	SWU11889	SWU11887	3	SWU12783	SWU12819	8.20	4.93	0.53	-0.07	-0.03	0.02	0.01
	1	SWU10912	DPL0090	4	BNL530	SWU21485	5.97	3.41	0.83	-0.06	-0.03	0.04	-0.01
	1	SWU0077	HAU1417	5	NAU6240	PGML1671	5.08	3.51	0.05	-0.06	-0.01	0.01	0.00
	4	**SWU16783**	**NAU3868**	5	PGML1671	PGML1917	6.11	3.77	0.70	0.06	0.04	-0.02	-0.02
	3	SWU12783	SWU12819	6	SWU19541	CGR5801	6.54	4.06	0.52	0.07	0.03	-0.02	-0.01
	2	SWU12025	SWU11889	7	CGR5001	CGR6586	8.43	4.71	0.90	-0.07	-0.03	0.04	-0.01
	8	HAU3177	NAU4064	13	DPL0894	SWU10800	5.84	3.05	0.98	0.06	0.02	-0.05	0.03
	5	SWU20917	NAU6240	14	NAU874	SWU13824	7.28	4.83	0.17	0.07	0.01	-0.02	0.01
	3	HAU2424	CER0028	15	DPL0182	SWU11691	5.17	3.71	0.03	0.06	0.00	-0.01	0.01
	5	PGML1917	SWU17715	16	NAU2984	SWU10062	9.06	4.74	1.03	0.07	0.04	-0.05	0.01
	14	SWU13909	TMB0071	16	SWU10038	ICR00016	5.40	3.55	0.29	0.06	0.02	-0.02	0.00
	5	CGR5025	NBRI0694	17	ICR03391	SWU12838a	5.94	4.26	0.03	-0.07	0.00	0.01	0.00
	8	CGR6508	Gh197	17	SWU12818	CGR5576	5.95	4.02	0.22	0.07	-0.01	-0.01	0.02
	13	SWU22374	HAU2857	18	SWU22281	SWU21800	6.36	4.04	0.25	-0.07	0.01	0.02	-0.02
	17	CGR5576	NAU3765	19	**NAU5330**	**Gh72**	5.44	3.18	0.68	0.06	0.03	-0.03	0.00
	16	CGR6802	HAU1129	19	NAU1042	NAU3437	5.02	3.36	0.13	0.06	0.00	0.02	-0.01
	5	NAU6240	PGML1671	19	NAU3437	NAU2894	5.15	3.35	0.12	0.06	0.01	-0.01	0.01
	7	SWU10785	CER0036	19	SWU17897	CGR5539	7.87	5.16	0.22	0.08	0.02	-0.01	-0.01
	17	ICR03391	SWU12838	20	CGR5548	SWU20675	5.00	3.05	0.08	-0.06	0.00	0.01	-0.01
	10	Gh320	HAU0635	20	SWU20636	CGR6154	5.80	4.35	0.04	0.07	0.01	0.00	0.00
	9	Gh27	SWU15194	20	SWU1259	SWU20033	5.20	3.42	0.25	-0.06	-0.02	0.02	0.00
	8	**DC20094**	**HAU1470b**	21	SWU16651	SWU16645	8.55	5.00	0.63	-0.07	-0.03	0.04	-0.01
	5	PGML1917	SWU17715	21	SWU16488	SWU16138	7.62	3.57	1.18	-0.06	-0.03	0.05	-0.03
	12	HAU1316	NAU3519	22	SWU21586	PGML1712	6.08	3.89	0.36	0.07	-0.01	-0.02	0.03
	3	SWU12732	SWU12783	24	CGR5202	Gh298	5.43	2.84	0.92	0.06	0.03	-0.04	0.01
	7	CGR5001	CGR6586	24	Gh298	SWU13133	6.11	2.23	1.37	0.05	0.01	-0.05	0.04
	11	CGR6580	SWU15972	24	SWU13267	BNL1521	5.35	3.08	0.70	0.06	0.02	-0.04	0.01
	6	CGR5108	ICR03206	24	BNL1521	HAU2504	5.37	2.17	1.14	0.05	0.01	-0.05	0.04
	12	DPL0732	Gh631	24	SWU13745	Gh273	6.09	3.76	0.61	-0.06	-0.02	0.04	-0.02
	11	NAU1014	ICR10344	25	SWU19129	PGML2858	5.09	3.60	0.14	-0.06	-0.01	0.02	0.00
	16	PGML1709	SWU10627	25	SWU19129	PGML2858	6.74	4.12	0.62	-0.07	-0.04	0.02	0.02
	14	SWU14224	DPL0565	25	SWU19411	SWU19412	5.63	3.48	0.54	0.06	0.03	-0.02	-0.01
	17	SWU12818	CGR5576	26	**DPL0491**	**Gh64**	5.33	3.16	0.56	-0.06	-0.02	0.04	-0.01
	26	SWU17395	DC30107	26	**DPL0491**	**Gh64**	5.88	3.92	0.03	-0.07	-0.01	0.01	0.00
	21	CGR5806	DPL0777	26	**DPL0491**	**Gh64**	5.52	3.91	0.03	-0.07	0.00	0.00	0.00
	25	Gh220	SWU19434	26	Gh64	SWU17257	5.83	3.52	0.38	-0.06	0.01	0.02	-0.03
	1	CGR6129	DPL0790	26	SWU17233	SWU17251	5.77	2.89	1.10	0.06	0.04	-0.05	0.01
	19	**TMB0107**	**NAU3217**	26	SWU18681	SWU0598	5.56	3.48	0.08	-0.06	-0.01	0.01	0.00
	26	BNL598	PGML1637	27	SWU10994	HAU1001	5.57	3.33	0.49	-0.06	-0.03	0.03	0.01
	7	SWU10205	HAU1483a	27	SWU10994	HAU1001	5.77	3.98	0.03	0.07	0.00	-0.01	0.00
	16	CGR6802	HAU1129	28	SHIN0219	TMB2386	5.89	3.72	0.36	-0.06	-0.02	0.03	-0.01
	4	SWU18876	SWU12672	28	SHIN0219	TMB2386	5.30	3.10	0.38	0.06	0.02	-0.03	0.00
	19	**NAU5330**	**Gh72**	28	SHIN0219	TMB2386	5.34	3.20	0.13	0.06	0.01	-0.02	0.00
	19	NAU3437	NAU2894	29	DC20127	DPL0252	5.90	3.86	0.22	0.07	0.02	-0.02	0.00
RILV population													
FL	7	C2_0046	HAU1367	9	MUSS139	PGML2830	5.29	4.06	0.03	-0.28	-0.02	0.02	-0.01
	7	CGR5372	C2_0046	12	BNL3261	Gh568	5.16	3.09	0.43	-0.25	0.10	-0.12	0.02

Trait	Chi	Flanking markers		Chj	Flanking markers		LOD	V(AA)	V(AAE)	AA	AAE1	AAE2	AAE3
	9	PGML2830	DC30015	12	SWU17197	Gh631	5.21	3.79	0.16	0.27	0.06	-0.08	0.01
	1	ICR03295	SWU10912	12	DPL0400	HAU2173	5.33	3.99	0.11	0.28	-0.07	0.04	0.02
	10	NAU3395	CAU0234	12	DPL0400	HAU2173	6.81	4.37	0.45	-0.30	0.10	-0.13	0.03
	14	HAU0883	CIR228	14	ICR01124	HAU2482	5.02	3.74	0.01	-0.33	-0.02	0.01	0.00
	6	MUSB1144	BNL3650	14	ICR03943	ICR12281	8.11	6.26	0.03	0.38	-0.04	0.03	0.01
	6	DPL0847	CGR6749	16	HAU3081	NAU747	5.36	4.00	0.04	-0.28	-0.04	0.03	0.00
	4	BNL530	SWU16781	20	Gh451	HAU1314	7.65	5.33	0.09	-0.32	0.05	-0.05	0.00
	6	BNL3650	TMB2940	21	**BNL1552**	**CGR5148**	5.42	3.82	0.12	-0.27	0.04	0.03	-0.07
	21	BNL3171	HAU2937	23	SHIN1076	BNL3482	5.98	4.69	0.02	0.31	-0.04	0.03	0.01
	1	SWU14514	Gh120	23	Gh327	ICR06429	5.14	3.66	0.09	-0.29	-0.01	-0.05	0.06
	10	NAU4967	SWU19932	23	MUSB994	NAU2238	6.13	4.26	0.12	-0.30	0.04	-0.04	0.00
	23	NAU2140	DC40286	23	MUSB994	NAU2238	5.18	3.62	0.00	-0.30	-0.04	-0.03	0.08
	9	MUSS139	PGML2830	24	PGML1207	Gh54	5.42	3.57	0.16	0.27	-0.03	0.09	-0.06
	10	SWU19932	HAU0635	24	PGML1207	Gh54	5.20	3.80	0.00	-0.27	0.02	-0.02	0.00
	14	ICR03943	ICR12281	24	PGML1207	Gh54	5.09	3.73	0.08	0.28	0.00	-0.05	0.05
	21	SWU16361	SWU16408	25	SWU19412	NAU3112	5.08	3.45	0.08	-0.26	0.02	0.04	-0.06
	16	HAU1129	C2_0011B	25	NAU4964	HAU1355	5.17	3.36	0.40	0.26	-0.11	0.11	0.00
	23	PGML1434	MUSB994	25	NAU4964	HAU1355	6.45	4.48	0.19	0.30	-0.06	0.09	-0.03
	14	DPL0502	ICR00401	26	MGHES31	HAU1571	5.24	3.42	0.27	-0.27	0.03	-0.10	0.07
	16	HAU3081	NAU747	29	DPL0171	Gh499	5.35	3.30	0.41	-0.26	0.11	-0.11	0.00
	10	NAU4967	SWU19932	29	DPL0171	Gh499	5.16	2.66	0.91	0.23	-0.10	0.19	-0.09
	4	BNL1167	JESPR234	29	Gh111	Gh27	7.15	5.12	0.02	0.32	-0.03	0.02	0.01
	6	DC40417	MUSB116	30	TMB1638	CGR6812	6.71	4.57	0.14	0.30	-0.06	0.07	-0.01
	21	BNL3171	HAU2937	31	NAU3109	CGR6772	5.41	4.39	0.03	-0.30	0.03	0.01	-0.03
	4	BNL1167	JESPR234	36	CGR5548	SWU20700	6.19	4.03	0.36	-0.29	0.07	-0.12	0.05
	9	MUSS139	PGML2830	36	CER0167	SWU20658	8.93	6.00	0.09	-0.35	0.05	0.01	-0.05
	14	ICR03943	ICR12281	36	CER0167	SWU20658	5.04	3.51	0.16	0.28	-0.07	0.07	0.00
	14	ICR01124	HAU2482	37	BNL5602	JESPR251	6.16	4.70	0.05	-0.37	-0.05	0.02	0.03
	31	SWU16780	SWU16735	37	BNL5602	JESPR251	9.21	6.48	0.03	0.36	-0.04	0.03	0.01
	6	DPL0847	CGR6749	38	**NAU2450**	**PGML1942**	6.54	4.53	0.29	-0.31	0.07	-0.11	0.04
	21	SWU16361	SWU16408	39	DPL0270	SWU16437	5.23	3.29	0.35	0.25	-0.10	0.10	-0.01
	23	HAU0244	Gh327	39	**SWU16437**	**SWU16432**	5.28	3.47	0.05	0.29	0.01	-0.06	0.05
FU	6	MUSB1144	BNL3650	10	NAU3395	CAU0234	6.11	3.52	0.53	-0.18	-0.10	0.04	0.06
	10	HAU0635	PGML4154	12	BNL3261	Gh568	5.35	2.30	0.90	-0.15	-0.02	-0.10	0.12
	20	SWU20027	Gh187	25	DPL0377	SWU19413	5.02	2.34	1.00	-0.15	0.09	-0.13	0.04
	16	HAU3081	NAU747	25	NAU4964	HAU1355	5.16	3.37	0.05	0.18	0.02	-0.02	0.00
	21	SWU16361	SWU16408	29	Gh111	Gh27	5.13	2.73	0.63	-0.16	-0.09	-0.01	0.10
	26	HAU1571	CGR6477	38	NAU2450	PGML1942	5.63	1.86	2.27	0.14	0.19	-0.15	-0.04
FS	9	MUSS139	PGML2830	10	SWU19932	HAU0635	5.28	2.74	0.48	0.27	0.14	0.00	-0.14
	9	CGR6876	BNL1317	12	HAU3373	CGR6847	5.02	2.13	0.82	0.24	0.13	0.07	-0.21
	10	NAU3395	CAU0234	12	DPL0400	HAU2173	5.29	2.24	1.26	-0.24	0.09	-0.26	0.17
	1	ICR03295	SWU10912	14	ICR00401	ICR03105	7.07	3.95	0.47	-0.40	-0.04	-0.15	0.19
	14	HAU2482	NAU4045	15	NAU3736	SWU11691	5.55	3.24	0.33	-0.36	0.08	-0.16	0.08
	6	MUSB1144	BNL3650	17	HAU1413	CGR5576	5.96	3.38	0.32	0.30	0.02	0.10	-0.12
	2	SWU12490	DPL0200	18	SWU21718	SWU0738	5.67	3.56	0.18	-0.31	-0.04	-0.06	0.10
	10	SWU13030	NAU4967	18	SWU0738	ICR02849	6.12	3.12	0.48	0.29	0.12	0.04	-0.15
	1	SWU14514	Gh120	19	SWU17789	SWU17882	5.43	3.54	0.05	0.31	0.05	-0.02	-0.03
	10	SWU19932	HAU0635	20	HAU1314	SWU20035	5.02	2.95	0.24	0.28	0.03	0.08	-0.11
	1	Gh529	SWU17434	21	**BNL1552**	**CGR5148**	5.70	3.24	0.20	-0.30	-0.04	-0.07	0.11

Trait	Chi	Flanking markers		Chj	Flanking markers		LOD	V(AA)	V(AAE)	AA	AAE1	AAE2	AAE3
	21	BNL3171	HAU2937	23	BNL3482	HAU0244	5.32	2.86	0.34	0.29	0.05	0.09	-0.14
	17	HAU1413	CGR5576	23	PGML1434	MUSB994	6.33	3.80	0.29	0.32	0.12	-0.05	-0.08
	23	SHIN0272	NAU2140	23	PGML1434	MUSB994	6.90	2.98	0.54	-0.33	-0.19	0.02	0.17
	14	DPL0502	ICR00401	24	Gh454	HAU3076	5.54	3.52	0.13	0.31	0.05	0.03	-0.08
	18	SWU0738	ICR02849	24	HAU3076	SWU13121	7.83	4.04	0.88	0.33	0.21	-0.04	-0.16
	16	HAU3081	NAU747	25	SWU19412	NAU3112	6.08	3.45	0.24	0.30	0.04	0.07	-0.11
	23	PGML1434	MUSB994	25	NAU4964	HAU1355	5.64	3.05	0.64	0.29	-0.04	0.18	-0.14
	1	SWU21958	NAU0748	29	Gh111	Gh27	8.20	4.46	0.70	0.35	0.07	0.12	-0.19
	21	SWU16361	SWU16408	30	TMB1638	CGR6812	5.19	3.21	0.26	0.29	0.10	0.00	-0.10
	18	SWU21718	SWU0738	34	DPL0897	SWU20341	5.34	3.35	0.25	-0.30	0.05	-0.12	0.07
	1	SWU14616	SWU14077	35	NAU2139	TMB1152	5.63	3.08	0.58	-0.29	0.03	-0.16	0.14
	9	MUSS139	PGML2830	36	CER0167	SWU20658	7.14	4.65	0.18	-0.35	0.04	-0.09	0.06
	26	MGHES31	HAU1571	38	NAU2450	PGML1942	5.18	2.71	0.57	0.27	0.09	0.08	-0.17
	21	**CGR5748**	**PGML250**	39	NAU5480	DPL0270	5.18	3.13	0.18	-0.29	-0.04	-0.07	0.11
FE	31	SWU16721	SWU16680	34	JESPR297	ICR00647	6.24	6.43	0.24	0.04	0.01	-	-0.01
FM	1	ICR03724	SWU11632	5	Gh260	PGML0120	5.38	3.57	0.02	0.07	0.00	-0.01	0.01
	2	**DPL0041**	**SWU12490**	7	HAU2530	CGR6586	6.20	4.04	0.14	0.07	0.02	0.00	-0.02
	6	BNL3650	TMB2940	7	HAU1367	NAU3181	5.27	3.20	0.33	-0.06	0.00	0.03	-0.02
	6	DPL0590	NAU2971	8	**HAU0810**	**TMB2904**	5.20	3.42	0.02	0.07	0.00	-0.01	0.00
	6	DPL0847	CGR6749	9	MUSS139	PGML2830	6.19	3.97	0.08	0.07	-0.02	0.01	0.00
	2	**DPL0041**	**SWU12490**	12	BNL3261	Gh568	5.66	3.77	0.05	-0.07	0.01	-0.01	0.00
	4	JESPR234	BNL530	12	ICR03107	HAU3373	5.03	2.74	0.32	-0.06	-0.02	0.03	-0.01
	6	SWU19656	CGR5124	12	HAU3373	CGR6847	5.26	3.56	0.03	-0.07	0.01	0.00	-0.01
	12	HAU3373	CGR6847	12	**SWU17197**	**Gh631**	5.76	3.38	0.00	0.07	-0.01	0.01	0.00
	1	**NAU0748**	**NAU2697**	12	DPL0400	HAU2173	6.34	4.17	0.02	0.07	0.00	0.00	-0.01
	12	Gh243	DPL0400	13	SWU13032	HAU2850	5.51	3.64	0.06	0.07	-0.01	0.01	0.00
	1	SWU14514	Gh120	13	SWU13032	HAU2850	5.01	3.42	0.04	-0.07	0.00	-0.01	0.01
	13	CER0165	SWU13032	14	ICR01124	HAU2482	6.36	4.15	0.08	-0.09	0.00	0.02	-0.01
	1	CGR6784	NAU3393	14	ICR03943	ICR12281	5.62	3.51	0.09	-0.07	0.02	0.00	-0.01
	12	HAU3373	CGR6847	15	**NAU3736**	**SWU11691**	5.76	3.84	0.03	0.07	-0.01	0.00	0.01
	14	ICR03105	ICR01124	16	**HAU1129**	**C2_0011B**	6.42	4.04	0.05	0.09	0.01	0.00	-0.01
	16	SWU18366	SWU18579	20	Gh451	HAU1314	5.29	3.55	0.01	0.07	0.00	0.00	0.00
	17	**HAU1413**	**CGR5576**	20	HAU1314	SWU20035	6.72	4.46	0.06	0.07	0.01	0.01	-0.01
	6	DPL0590	NAU2971	21	CGR6521	Gh450	5.82	3.78	0.07	0.07	0.00	-0.01	0.01
	12	HAU3373	CGR6847	21	CGR6521	Gh450	5.78	3.49	0.20	0.07	-0.01	-0.01	0.02
	10	**SWU19932**	**HAU0635**	23	DC40286	PGML1434	5.39	3.56	0.00	0.07	0.00	0.00	0.00
	23	SHIN0272	NAU2140	23	PGML1434	MUSB994	5.95	4.36	0.14	0.08	0.00	0.02	-0.01
	4	BNL1167	JESPR234	25	BNL3098	HAU1224	5.27	3.44	0.07	-0.07	-0.01	0.01	-0.01
	16	SWU18366	SWU18579	25	BNL3098	HAU1224	7.10	4.49	0.17	0.08	-0.02	0.01	0.01
	21	CGR6521	Gh450	31	DPL0057	NAU3109	5.60	3.45	0.22	0.07	0.01	-0.02	0.02
	4	SWU16783	SWU18876	31	SWU16780	SWU16735	5.35	3.36	0.05	-0.07	0.00	-0.01	0.01
	1	CGR6784	NAU3393	31	SWU16730	SWU16721	7.37	4.84	0.05	-0.08	-0.01	0.01	0.00
	31	SWU16730	SWU16721	31	SWU16721	SWU16680	5.27	3.37	0.25	-0.07	0.00	0.02	-0.02
	8	**HAU0810**	**TMB2904**	35	NAU2139	TMB1152	5.28	3.32	0.02	-0.07	0.00	0.00	0.00
	32	HAU1000	TMB1931	37	HAU0423	JESPR154	5.87	2.89	0.98	0.06	-0.02	-0.03	0.05
	25	NAU2968	DPL0377	37	BNL5602	JESPR251	5.33	3.02	0.44	-0.06	0.01	0.02	-0.03
	30	TMB1638	CGR6812	38	NAU2450	PGML1942	5.15	3.19	0.23	-0.06	0.00	0.02	-0.02
	31	SWU16735	SWU16755	39	NAU5480	DPL0270	5.55	3.45	0.24	0.07	0.00	-0.02	0.02
	14	HAU0883	CIR228	39	DPL0270	SWU16437	6.50	3.92	0.30	0.07	-0.03	0.01	0.02

Trait	Chi	Flanking markers		Chj	Flanking markers		LOD	V(AA)	V(AAE)	AA	AAE1	AAE2	AAE3
	37	BNL5602	JESPR251	39	SWU16437	SWU16432	5.78	3.60	0.24	-0.07	0.01	0.01	-0.03

Chi and Chj represent the group number of the loci being tested in the analysis

Flanking markers in bold are those flanking M-QTLs identified

AA is the epistatic effect between loci i and j

AAE is the effect of the environmental interaction of epistasis

AAEl, AAE2 and AAE3 indicate the epistatic effects of QTL × environment interactions in E1, E2 and E3, respectively

V(AA)% and V(AAE)%, percentage of the total variation explained by the AA and AAE
