# Supplementary material for: Genetic Analysis and QTL Detection on Fiber Traits Using Two Recombinant Inbred Lines and Their Backcross Populations in Upland Cotton
Source: G3 (Bethesda). 2016 Jun 23;6(9):2717–24. doi: 10.1534/g3.116.031302 (PMC5015930; doi:10.1534/g3.116.031302)
Supplement: Supplemental Material [file supp_g3.116.031302_TableS6.pdf]

Table S6 Epistatic effects and environmental interactions detected for fiber quality traits in BCF_1 and $BCVF_1$ populations using two-locus analysis by ICIMapping 4.0

Trait	Chi	Flanking markers		Chj	Flanking markers		LOD	V(AA)	V(AAE)	AA	AAE1	AAE2	AAE3
BCF_1 population													
FL	1	NAU6367	MUSS422	3	SWU12819	SWU12765	5.50	2.05	2.15	0.10	-0.10	0.15	-0.05
	1	NAU3177	ICR03724	11	NAU3390	NAU2460	6.68	1.50	4.04	-0.09	0.13	-0.21	0.07
	11	CGR5421	ICR08245	11	ICR01810	CGR6525	5.50	2.24	1.91	0.12	-0.08	0.16	-0.08
	5	DPL0022	SWU17787	11	SWU15972	TMB0628	5.45	3.46	0.39	-0.14	-0.01	-0.05	0.06
	1	SWU10986	NAU2218	19	NAU1042	NAU3437	5.41	3.10	0.58	-0.13	0.00	-0.07	0.07
	10	**Gh320**	**HAU0635**	20	SWU20675	SWU20649	5.92	3.48	0.79	0.14	-0.07	0.09	-0.02
	6	ICR10602	SWU19656	22	SWU21646	SWU21585	6.42	3.67	0.85	0.14	-0.07	0.09	-0.02
	1	NAU3384	CGR5663	23	PGML4186	NAU3100	5.79	3.42	0.77	-0.13	0.00	-0.08	0.09
	21	SWU14431a	SWU15915	24	BNL1521	HAU2504	5.82	2.53	1.72	-0.12	0.06	-0.14	0.08
	5	**SWU20917**	**NAU6240**	24	SWU13758	CGR5423	5.52	3.39	0.26	0.13	0.04	0.01	-0.05
	5	**SWU20917**	**NAU6240**	25	CGR6864	SWU19815	7.42	3.84	1.08	-0.14	-0.01	-0.09	0.10
	6	HAU1460	HAU1371	25	SWU19763	SWU19129	5.57	3.21	0.80	0.13	-0.05	0.09	-0.04
	14	SWU14224	DPL0565	26	BNL2495	DPL0491	5.91	3.61	0.28	-0.14	0.01	-0.05	0.04
	19	NAU3437	NAU2894	26	DPL0491	Gh64	5.18	3.17	0.43	-0.13	0.01	-0.07	0.06
	1	SWU11191	BNL2827b	29	C2_0115	ICR03107	5.78	2.51	1.72	-0.12	0.10	-0.13	0.03
	6	ICR03206	NAU896	29	C2_0115	ICR03107	5.46	2.31	1.90	-0.11	0.08	-0.14	0.06
FU	11	BNL3442b	ICR01810	21	BNL3171	CGR5808	5.57	1.42	1.38	0.11	0.06	0.09	-0.15
	18	CIR099	NAU748	27	SWU10994	HAU1001	5.70	1.65	2.50	0.11	-0.07	0.20	-0.13
	14	TMB0071	BNL3661	27	ICR11205	DPL0847a	5.22	1.08	2.69	-0.10	0.13	-0.19	0.06
	3	SWU12819	SWU12765	29	C2_0115	ICR03107	5.19	1.59	1.50	0.11	-0.02	0.14	-0.12
	21	CGR5602	JESPR154	32	NAU2140	NAU2957	5.57	3.04	0.63	0.16	0.08	0.01	-0.09
FS	4	SWU16782	SWU16783	6	CGR5801	SWU19249	5.25	1.97	1.15	0.12	0.13	-0.04	-0.09
	9	HAU190	HAU1618	13	DPL0894	SWU10800	5.22	1.18	2.08	0.10	0.03	0.14	-0.17
	4	SWU16782	SWU16783	20	SWU1259	SWU20033	5.06	3.24	0.35	0.16	0.06	0.00	-0.06
	22	DPL0562	CAU0161	25	SWU19848	CGR6864	5.36	1.60	1.32	0.12	0.04	0.11	-0.15
	21	CGR5217	BNL3442a	26	BNL598	PGML1637	6.04	1.26	2.29	0.10	0.00	0.16	-0.17
	3	SWU12765	NAU3839	26	SWU17336	NAU5072	5.04	2.45	0.44	0.14	-0.04	0.08	-0.05
	13	**NAU3468**	**SWU22309**	27	SWU11038	SWU11384	5.73	2.16	1.51	0.13	0.15	-0.09	-0.06
FE	13	DPL0894	SWU10800	18	DC40150	ICR02849	5.89	1.82	4.27	0.02	0.02	-0.02	-
FM	1	NAU3384	CGR5663	2	**SWU11887**	**SWU11976**	6.64	3.69	0.28	-0.04	-0.01	-0.01	0.02
	1	ICR03724	ICR03725	4	ICR01729	SWU16781	5.12	2.31	0.59	-0.03	-0.01	-0.01	0.02
	5	SWU20913	Gh260	6	HAU1460	HAU1371	5.56	3.64	0.15	-0.04	-0.01	0.00	0.01
	5	SWU17787	SWU13378	11	NAU3390	NAU2460	6.00	3.42	0.70	-0.04	-0.02	0.00	0.02
	7	CGR5372	SWU10205	11	NAU3390	NAU2460	5.63	2.82	1.16	0.04	0.00	0.03	-0.03
	5	HAU1603	PGML4457	13	DPL0535	CER0165	5.02	3.03	0.49	-0.04	0.00	-0.02	0.02
	10	SWU20501b	CGR5873	14	**PGML1568**	**Gh529**	5.25	3.01	0.66	0.04	-0.01	0.02	-0.02
	6	HAU1460	HAU1371	14	NAU3820	NAU2960	6.13	2.85	1.15	0.04	0.02	0.01	-0.03
	1	SWU11191	BNL2827b	16	PGML1709	SWU10627	6.50	2.22	2.28	0.03	-0.02	0.05	-0.03
	4	**SWU16783**	**NAU3868**	16	SWU10214	Gh56	5.13	1.35	1.85	-0.03	-0.01	-0.03	0.04
	5	SWU20917	NAU6240	16	Gh56	NAU5120	7.58	2.86	2.04	0.04	0.02	0.02	-0.04
	13	SHIN1462	SWU22374	16	SWU10062	SWU10094	6.60	3.87	0.44	-0.04	-0.01	-0.01	0.02
	9	Gh158	DC40407	18	CIR099	NAU748	5.92	3.14	0.77	0.04	0.00	0.02	-0.03
	1	NAU3384	CGR5663	18	DC40150	ICR02849	5.44	3.66	0.43	-0.04	0.02	-0.02	0.00
	5	SWU13378	SWU17846	19	SWU17782	DPL0056	5.87	1.81	1.69	0.03	0.00	0.03	-0.04

Trait	Chi	Flanking markers		Chj	Flanking markers		LOD	V(AA)	V(AAE)	AA	AAE1	AAE2	AAE3
	7	NAU1357	SWU10067	19	**TMB0107**	**NAU3217**	6.03	3.04	1.03	0.04	0.01	0.02	-0.03
	3	SWU12783	SWU12819	21	SWU0830	HAU2004	5.27	1.76	1.44	0.03	0.02	0.02	-0.04
	1	NAU3177	ICR03724	21	SWU14431a	SWU15915	5.61	0.84	2.09	0.02	0.03	0.02	-0.05
	14	NAU2960	ICR12130	21	SWU15915	SWU0189	5.81	3.69	0.40	0.04	0.01	0.01	-0.02
	21	Gh451	SWU16489	22	PGML0695	SWU20813	5.21	2.85	0.48	-0.04	-0.01	-0.01	0.02
	1	BNL2827a	NAU6367	23	PGML4186	NAU3100	5.23	2.84	0.46	0.04	0.02	0.00	-0.02
	3	SWU12783	SWU12819	23	PGML4186	NAU3100	5.05	2.88	0.34	0.04	0.01	0.01	-0.02
	13	SWU13032	DPL0308	24	SWU13267	BNL1521	6.03	3.25	0.81	-0.04	-0.01	-0.02	0.03
	6	CGR5801	SWU19249	24	SWU13745	Gh273	5.17	2.79	0.74	0.04	-0.01	0.03	-0.02
	16	SWU10627	PGML1309	25	SWU19144	Gh220	5.53	2.95	0.59	-0.04	0.00	-0.02	0.02
	2	SWU11950	TMB1268	25	Gh220	SWU19434	5.34	2.10	1.41	0.03	0.01	0.02	-0.04
	7	SWU10067	SWU10064	26	MGHES31	HAU1571	5.61	1.83	1.88	0.03	0.02	0.02	-0.04
	3	SWU12819	SWU12765	26	SWU17395	DC30107	5.07	2.39	0.86	-0.03	-0.01	-0.02	0.03
	24	SWU13758	CGR5423	26	NAU2175	SWU17336	6.02	1.28	1.94	-0.03	-0.03	-0.02	0.04
	20	SWU20700	CGR5548	27	SWU11384	ICR11885	6.06	3.41	1.04	-0.04	-0.02	-0.02	0.03
	13	PGML0014	CGR6732	28	BNL2877	HAU3071	5.15	1.75	1.64	-0.03	-0.02	-0.02	0.04
	4	SWU18881	NAU2701	28	SHIN0219	TMB2386	5.11	2.54	0.98	0.04	0.03	0.00	-0.03
	19	NAU3437	NAU2894	29	DC20127	DPL0252	5.46	2.51	1.10	0.03	0.03	-0.01	-0.03
	12	DPL0303	COT107	29	DC20127	DPL0252	5.48	3.14	0.40	-0.04	-0.01	-0.01	0.02
	21	SWU14431a	SWU15915	29	C2_0115	ICR03107	5.08	2.84	0.57	-0.04	-0.02	0.01	0.02
	10	NAU2139	SWU20689	30	BNL243	CER0168	5.10	2.71	0.55	-0.04	0.01	-0.02	0.01
	5	CGR5025	NBRI0694	30	CER0168	SWU21718	5.18	2.99	0.58	0.04	-0.01	0.03	-0.01
	26	SWU17233	SWU17251	31	SWU16676	SWU16755	6.38	3.93	0.48	-0.04	-0.01	-0.01	0.02
					BCVF$_1$ population								
FL	6	SWU19656	CGR5124	6	CGR6749	NAU3186	6.02	1.17	1.97	-0.10	-0.08	-0.10	0.18
	6	CGR5355	SWU19656	14	ICR03943	ICR12281	6.91	4.10	0.24	0.19	0.02	0.04	-0.06
	13	CER0165	SWU13032	16	HAU3081	NAU747	5.86	3.42	0.99	0.16	-0.04	0.12	-0.08
	3	SWU12840	NAU2742	20	HAU1314	SWU20035	5.25	2.94	0.13	-0.15	0.02	-0.05	0.04
	21	BNL3171	HAU2937	23	BNL3482	HAU0244	5.63	2.74	0.61	0.15	0.00	0.09	-0.08
	16	SWU10266	SWU18366	23	BNL3482	HAU0244	5.24	2.77	0.39	0.15	0.01	0.07	-0.07
	19	SWU17789	SWU17882	23	MUSB994	NAU2238	5.39	2.77	0.58	0.15	0.04	0.05	-0.10
	14	ICR03943	ICR12281	24	PGML1207	Gh54	5.31	2.54	0.84	0.15	-0.01	0.11	-0.10
	2	DPL0200	SWU11889	31	SWU16721	SWU16680	5.22	3.21	0.04	0.16	0.02	0.00	-0.02
	2	**SWU11976**	**SWU12001**	34	JESPR297	ICR00647	5.27	2.22	1.13	-0.13	0.02	-0.12	0.10
	23	BNL3482	HAU0244	37	**HAU0423**	**JESPR154**	5.41	2.56	0.69	-0.15	-0.01	-0.09	0.10
	31	SWU16780	SWU16735	37	BNL5602	JESPR251	6.13	3.78	0.06	0.17	0.02	0.01	-0.03
	20	SWU20246	Gh451	38	NAU2450	PGML1942	5.82	3.38	0.13	-0.16	-0.04	0.00	0.04
FS	1	SWU14616	SWU14077	9	PGML2830	DC30015	5.73	2.83	0.62	0.17	0.11	-0.03	-0.08
	6	CGR5355	SWU19656	14	ICR03943	ICR12281	5.51	2.46	1.13	0.17	0.00	0.14	-0.14
	6	MUSB1144	BNL3650	21	**BNL1552**	**CGR5148**	5.11	3.07	0.43	-0.18	-0.09	0.01	0.08
	16	NAU747	HAU1129	23	SHIN0272	NAU2140	5.98	3.76	0.24	-0.23	0.08	-0.09	0.01
	16	HAU3081	NAU747	25	NAU4964	HAU1355	5.58	3.45	0.39	0.19	0.07	0.02	-0.09
	2	SWU12490	DPL0200	31	SWU16730	SWU16721	5.06	2.83	0.23	0.17	0.03	0.04	-0.07
	9	SWU15517	MGHES06	38	NAU2450	PGML1942	5.40	3.52	0.24	-0.20	-0.04	-0.03	0.08
FM	12	HAU3373	CGR6847	12	SWU17197	Gh631	5.37	2.74	0.26	0.04	-0.01	0.02	0.00
	1	Gh398	CGR6129	18	SWU0738	ICR02849	5.06	0.09	2.92	0.01	-0.06	0.03	0.03
	1	SWU21958	NAU0748	23	**CGR5158**	**HAU1758**	5.48	3.37	0.00	-0.04	0.00	0.00	0.00
	6	CGR6749	NAU3186	29	Gh111	Gh27	6.03	3.39	0.20	-0.04	-0.01	0.01	-0.01
	4	BNL530	SWU16781	31	SWU16780	SWU16735	5.13	0.50	2.44	-0.02	0.05	-0.01	-0.04

Trait	Chi	Flanking markers		Chj	Flanking markers		LOD	V(AA)	V(AAE)	AA	AAE1	AAE2	AAE3
	24	PGML1207	Gh54	35	NAU2139	TMB1152	6.69	4.32	0.03	0.05	0.00	0.01	0.00
	1	SWU21958	NAU0748	36	CER0167	SWU20658	5.38	3.00	0.53	0.04	0.02	0.01	-0.02
	14	HAU0883	CIR228	39	DPL0270	SWU16437	5.07	2.73	0.43	0.04	-0.02	0.01	0.01

See footnotes of supplementary table S5 for explanations
