# Supplementary material for: Genetic Analysis and QTL Detection on Fiber Traits Using Two Recombinant Inbred Lines and Their Backcross Populations in Upland Cotton
Source: G3 (Bethesda). 2016 Jun 23;6(9):2717–24. doi: 10.1534/g3.116.031302 (PMC5015930; doi:10.1534/g3.116.031302)
Supplement: Supplemental Material [file supp_g3.116.031302_TableS7.pdf]

**Table S7** Same QTLs for fiber quality traits identified using composite interval mapping in previous RIL population (Shang et al. 2015a)

| Trait | QTL | Env. | Position | Marker interval | | LOD | A | Var% |
|---|---|---|---|---|---|---|---|---|
| Fiber length | ***qFL-Chr5-1*** | E1 | 39.31 | NAU6240 | PGML1671 | 2.87 | 0.27 | 7.61 |
| | | E2 | 26.91 | SWU20917 | NAU6240 | 3.95 | 0.39 | 11.02 |
| | | E3 | 14.71 | PGML0120 | SWU20914 | 2.94 | 0.21 | 5.61 |
| | ***qFL-Chr5-2*** | E2 | 114.11 | NAU4034 | SWU17713 | 2.92 | 0.29 | 5.65 |
| | | E1 | 114.11 | NAU4034 | SWU17713 | 6.33 | 0.33 | 11.59 |
| | *qFL-Chr5-3* | E3 | 120.71 | PGML4457 | MUSS193 | 4.40 | 0.27 | 8.97 |
| | *qFL-Chr5-4* | E3 | 130.81 | NBRI0694 | DPL0022 | 4.83 | 0.27 | 9.47 |
| | *qFL-Chr5-5* | E3 | 143.41 | SWU17787 | SWU13378 | 3.62 | 0.29 | 10.37 |
| | *qFL-Chr10-1* | E2 | 41.01 | SWU20260 | Gh144 | 3.93 | -0.33 | 7.78 |
| | *qFL-Chr18-1* | E1 | 117.21 | SWU22192 | DPL0864 | 3.72 | -0.25 | 6.58 |
| | *qFL-Chr21-1* | E1 | 171.11 | BNL3171 | CGR5808 | 3.97 | -0.27 | 7.74 |
| Fiber strength | ***qFS-Chr5-1*** | E1 | 26.91 | SWU20917 | NAU6240 | 4.27 | 0.45 | 9.99 |
| | | E2 | 26.91 | SWU20917 | NAU6240 | 4.73 | 0.43 | 12.58 |
| | | E3 | 26.91 | SWU20917 | NAU6240 | 4.91 | 0.34 | 11.57 |
| | ***qFS-Chr15-1*** | E3 | 4.71 | CGR6889 | DPL0182 | 3.06 | 0.24 | 5.64 |
| | | E2 | 6.71 | CGR6889 | DPL0182 | 2.76 | 0.29 | 5.48 |
| | ***qFS-Chr19-1*** | E3 | 40.51 | NAU3437 | NAU2894 | 4.61 | 0.40 | 15.54 |
| | | E1 | 36.51 | NAU3437 | NAU2894 | 3.47 | 0.40 | 7.84 |
| Fiber elongation | *qFE-Chr2-1* | E1 | 84.51 | SWU12343 | SWU14060 | 5.99 | 0.06 | 11.63 |
| | *qFE-Chr5-1* | E1 | 114.11 | NAU4034 | SWU17713 | 5.40 | 0.06 | 10.35 |
| | *qFE-Chr5-2* | E3 | 119.41 | HAU1603 | PGML4457 | 3.60 | 0.04 | 8.13 |
| | *qFE-Chr6-1* | E3 | 49.51 | HAU1460 | HAU1371 | 5.33 | 0.04 | 10.70 |
| Micronaire | ***qFM-Chr2-1*** | E2 | 52.01 | SWU11887 | SWU11976 | 2.98 | -0.08 | 8.14 |
| | | E3 | 60.01 | SWU11976 | SWU11950 | 2.72 | -0.09 | 5.82 |
| | *qFM-Chr9-1* | E3 | 44.41 | SWU15194 | HAU190 | 4.59 | 0.18 | 25.93 |
| | ***qFM-Chr19-1*** | E3 | 4.01 | NAU5330 | Gh72 | 2.76 | 0.09 | 5.59 |
| | | E1 | 6.01 | NAU5330 | Gh72 | 2.55 | 0.08 | 5.14 |
| | *qFM-Chr26-1* | E1 | 35.91 | SWU16753 | SWU16780 | 3.43 | -0.10 | 6.81 |

Env. : Environment, E1, Handan; E2: Cangzhou; E3: Xiangyang

Bold figures indicate the QTL was detected in more than two environments simultaneously

A, Additive effect

Var%, Phenotypic variation explained by a single QTL
