# Supplementary material for: Genetic Analysis and QTL Detection on Fiber Traits Using Two Recombinant Inbred Lines and Their Backcross Populations in Upland Cotton
Source: G3 (Bethesda). 2016 Jun 23;6(9):2717–24. doi: 10.1534/g3.116.031302 (PMC5015930; doi:10.1534/g3.116.031302)
Supplement: Supplemental Material [file supp_g3.116.031302_TableS8.pdf]

Table S8 QTLs identified for fiber traits using the overall means by composite interval mapping

Trait	QTL	Marker interval		RIL			BC		
				LOD	A	Var%	LOD	A+D	Var%
		XZ hybrid							
FL	***qFL-Chr2-1***	SWU12025	SWU11889				2.97	0.14	5.93
	qFL-Chr2-2	SWU11977	SWU11951	3.03	0.22	6.27			
	qFL-Chr2-3	PGML0700	SWU12016	3.15	0.21	5.69			
	qFL-Chr5-1	SWU20917	NAU6240	3.67	0.26	8.98			
	qFL-Chr5-2	NAU4034	SWU17713	7.26	0.33	13.91			
		NAU4034	SWU17713				3.00	0.13	5.99
	qFL-Chr5-3	PGML4350	SWU17781	4.36	0.26	9.31			
	qFL-Chr10-1	ICR00093	ICR07050	4.31	-0.30	11.62			
	qFL-Chr10-2	Gh320	HAU0635	3.82	-0.26	9.23			
	qFL-Chr16-1	NAU5120	NAU862				4.67	-0.19	9.74
	qFL-Chr16-2	SWU10094	SWU10060				3.02	0.17	8.71
	qFL-Chr21-1	SWU15915	SWU0189				3.61	-0.15	7.30
	qFL-Chr26-1	CGR6254	SWU16676				2.67	0.12	4.94
FU	***qFU-Chr2-1***	NBRI0014	SWU12107				3.81	0.17	8.69
	qFU-Chr3-1	HAU2425	CER0029	2.76	-0.14	6.19			
	qFU-Chr26-1	CGR6254	SWU16676				3.78	0.16	7.72
FS	***qFS-Chr5-1***	SWU20917	NAU6240	7.06	0.44	18.11			
	qFS-Chr5-2	PGML1671	PGML1917				3.16	0.16	6.48
	qFS-Chr21-1	BNL3171	CGR5808	3.22	-0.26	6.28			
	qFS-Chr21-2	CGR5217	BNL3442a				3.50	-0.17	8.00
FE	***qFE-Chr2-1***	SWU12126	SWU12147	2.95	0.03	6.10			
	qFE-Chr2-2	SWU14060	NBRI0014				2.65	0.02	5.14
	qFE-Chr5-1	PGML1917	SWU17715				5.22	0.04	23.60
	qFE-Chr5-2	NAU4034	SWU17713				6.43	0.03	13.01
	qFE-Chr5-3	HAU1603	PGML4457	5.00	0.05	12.12			
	qFE-Chr5-4	PGML4350	SWU17781				3.15	0.02	7.30
	qFE-Chr13-1	DPL0572	HAU2558				4.47	0.02	9.19
	qFE-Chr18-1	CIR099	NAU748	2.73	0.03	5.28			
	qFE-Chr24-1	PGML4657	Gh454	3.49	-0.04	6.89			
FM	***qFM-Chr9-1***	SWU15194	HAU190	3.15	0.11	15.17			
	qFM-Chr12-1	CGR5111	C2_0115				3.83	0.06	8.33
	qFM-Chr14-1	PGML1568	Gh529	2.61	0.07	7.05			
	qFM-Chr15-1	DC40183	DC40175	3.25	0.07	6.23			
	qFM-Chr19-1	NAU5330	Gh72	3.12	0.07	6.00			
		Gh616	CIR139				3.29	0.04	7.27
	qFM-Chr26-1	NAU2175	SWU17336				3.75	-0.05	9.40
	qFM-Chr26-2	SWU17336	NAU5072				3.32	-0.04	7.58
	qFM-Chr26-3	SWU16676	SWU16755	2.77	-0.08	8.81			
		XZV hybrid							
FL	***qFL-Chr2-1***	SWU11976	SWU12001	5.49	0.41	10.63			
		SWU11976	SWU12001				3.70	0.21	8.87
	qFL-Chr14-1	HAU1000	TMB1931				4.03	0.21	8.19

Trait	QTL	Marker interval		RIL			BC		
				LOD	A	Var%	LOD	A+D	Var%
	qFL-Chr19-1	NAU2893	HAU3069	2.64	0.28	5.04			
	qFL-Chr23-1	NAU5373b	HAU2648	3.68	-0.37	7.15			
FU	***qFU-Chr18-1***	TMB1638	CGR6812				3.70	-0.03	14.39
FS	***qFS-Chr21-1***	CGR5748	PGML2500	2.90	-0.32	5.12			
	qFS-Chr23-1	NAU3588	NAU5373a	3.57	-0.36	6.78			
	qFS-Chr26-1	HAU1571	CGR6477	4.33	-0.42	9.47			
		CGR6477	PGML2562				4.56	-0.25	10.49
FE	***qFE-Chr14-1***	CIR228	DPL0502	2.84	0.03	5.87			
	qFE-Chr26-1	CGR6477	PGML2562	3.17	-0.04	7.72			
FM	*qFM-Chr2-1*	BNL3545	PGML3983				3.03	0.05	5.75
	qFM-Chr14-1	BNL3661	PGML4891				3.03	0.05	7.17
	qFM-Chr14-2	HAU1000	TMB1931	6.24	-0.12	14.12			
	qFM-Chr23-1	MUSB994	NAU2238				4.41	-0.07	11.43
	qFM-Chr23-2	NAU5373a	NAU5373b				3.92	-0.06	7.52
	qFM-Chr25-1	SWU19412	NAU3112	3.23	0.09	8.37			
	qFM-Chr25-2	NAU4964	HAU1355	3.63	0.08	7.01			

QTL identified from single environment and the overall means of three environments were compared, and common QTLs are shown as bold figures

Effect, the genetic expectation of a QTL effect obtained is the additive effect (A) when estimated from the RILs, the additive and dominance effects (A+D) from the BCF_1 mean values

Var%, Phenotypic variation explained by a single QTL
